# Hepatocellular carcinoma surveillance based on the Australian Consensus Guidelines: a health economic modelling study

**DOI:** 10.1186/s12913-023-09360-4

**Published:** 2023-04-19

**Authors:** Anh Le Tuan Nguyen, Lei Si, John S Lubel, Nicholas Shackel, Kwang Chien Yee, Mark Wilson, Jane Bradshaw, Kerry Hardy, Andrew John Palmer, Christopher Leigh Blizzard, Barbara de Graaff

**Affiliations:** 1 Menzies Institute for Medical Research, University of Tasmania, 17 Liverpool Street, Hobart, TAS 7000 Australia; 2 School of Health Sciences, Western Sydney University, Campbelltown, Australia; 3 Translational Health Research Institute, Western Sydney University, Penrith, Australia; 4 Alfred Health, Melbourne, VIC Australia; 5 Monash University, Melbourne, VIC Australia; 6 University of New South Wales, Sydney, NSW Australia; 7 School of Medicine, University of Tasmania, Hobart, TAS Australia; 8 Royal Hobart Hospital, Hobart, TAS Australia

**Keywords:** Hepatocellular carcinoma, Surveillance, Cost-effectiveness, Ultrasound, Alpha-fetoprotein

## Abstract

**Background:**

Hepatocellular carcinoma (HCC) is the fastest increasing cause of cancer death in Australia. A recent Australian consensus guidelines recommended HCC surveillance for cirrhotic patients and non-cirrhotic chronic hepatitis B (CHB) patients at gender and age specific cut-offs. A cost-effectiveness model was then developed to assess surveillance strategies in Australia.

**Methods:**

A microsimulation model was used to evaluate three strategies: biannual ultrasound, biannual ultrasound with alpha-fetoprotein (AFP) and no formal surveillance for patients having one of the conditions: non-cirrhotic CHB, compensated cirrhosis or decompensated cirrhosis. One-way and probabilistic sensitivity analyses as well as scenario and threshold analyses were conducted to account for uncertainties: including exclusive surveillance of CHB, compensated cirrhosis or decompensated cirrhosis populations; impact of obesity on ultrasound sensitivity; real-world adherence rate; and different cohort’s ranges of ages.

**Results:**

Sixty HCC surveillance scenarios were considered for the baseline population. The ultrasound + AFP strategy was the most cost-effective with incremental cost-effectiveness ratios (ICER) compared to no surveillance falling below the willingness-to-pay threshold of A$50,000 per quality-adjusted life year (QALY) at all age ranges. Ultrasound alone was also cost-effective, but the strategy was dominated by ultrasound + AFP. Surveillance was cost-effective in the compensated and decompensated cirrhosis populations alone (ICERs < $30,000), but not cost-effective in the CHB population (ICERs > $100,000). Obesity could decrease the diagnostic performance of ultrasound, which in turn, reduce the cost-effectiveness of ultrasound ± AFP, but the strategies remained cost-effective.

**Conclusions:**

HCC surveillance based on Australian recommendations using biannual ultrasound ± AFP was cost-effective.

**Supplementary Information:**

The online version contains supplementary material available at 10.1186/s12913-023-09360-4.

## Background

Primary liver cancer (PLC) is amongst the most deadly cancers, ranking second in the cause of cancer mortality globally [1]. The most common form of PLC is hepatocellular carcinoma (HCC), which accounts for more than 80% of total PLC cases [2]. In Australia, although mortality rates of many cancers have plateaued or reduced, cancer death due to HCC is rising [3], making it the fastest increasing cause of cancer mortality in this country [4].

The outcomes of HCC patients are highly dependent on tumour stage at diagnosis [5]. Those diagnosed at the early stages are more suitable candidates for curative treatments (liver resection, ablation, or transplant) than those diagnosed at later stages [6, 7]. However, HCC is infrequently detected early due to its asymptomatic nature at early stages [8–11]. In general, when the symptoms manifest, HCC has progressed to advanced stages [12–14]. A recent study in seven hospitals based in Melbourne, Australia found only 26% of people newly diagnosed with HCC were at an early stage of tumour development [15]. Similarly, other studies reporting on HCC in the USA [16] and Austria [17] reported less than 20% of patients were at an early stage when diagnosed.

Many professional bodies, including the American Association for the Study of Liver Diseases [18], the European Association for the Study of the Liver [19], and the Asian Pacific Association for the Study of the Liver [20] have recommended ultrasound with or without the biomarker Alpha-fetoprotein (AFP) at 6-month intervals as a HCC surveillance strategy to improve the early detection of HCC. An Australian consensus statement for the management of HCC was also published with a high level of agreement on using liver ultrasound with or without the combination of AFP at 6-month intervals for HCC surveillance with high-risk populations [21]. These high-risk populations consist of people having liver cirrhosis regardless of age and non-cirrhotic people with chronic hepatitis B (CHB) infection including Asian men older than 40 years, Asian women older than 50, Sub-Saharan African people older than 20, and Aboriginal and Torres Strait Islander people older than 50 [21].

Over the last few decades, many economic evaluations of HCC surveillance have been conducted. The studies used modelling techniques to evaluate the cost and outcomes of different surveillance strategies to inform decision making. A recent systematic review has shown most of these evaluations were cost-effective, but their results need to be interpreted with care due to limitations existing within those studies [4]. For that reason, we have developed a health economic evaluation model, based on the Australian consensus statement, that takes into account the limitations of previous models.

## Methods

### Study setting and surveillance strategies

In Australia, HCC is managed by offering possible curative intent whilst exposing patients to minimal risk with treatment [21]. It is also critical that patients understand their disease and clinicians respect patients’ choices. The Barcelona Clinic Liver Cancer (BCLC) staging system is recommended as the framework for HCC management in Australia [21]. It classifies HCC into five stages ranging from very early (0) to terminal (D) and links those stages with a suitable treatment algorithm [22].

For this model, current practice of HCC management in Australia was defined as no formal surveillance or the *status quo*. For the *status quo*, HCC is found either incidentally or when HCC becomes symptomatic. The *status quo* was compared with four other strategies: biannual ultrasound at real-world adherence rates [23]; biannual ultrasound with AFP at real-world adherence rates and both strategies at 100% (full) adherence rates. Due to the lack of real-world adherence data in Australia, the adherence rates were obtained from a USA-based study of HCC surveillance in a hepatitis B-infected Asian population [23] and set as calibration targets for the surveillance adherence rate in the model.

### Overview of the model

**Fig. 1:**
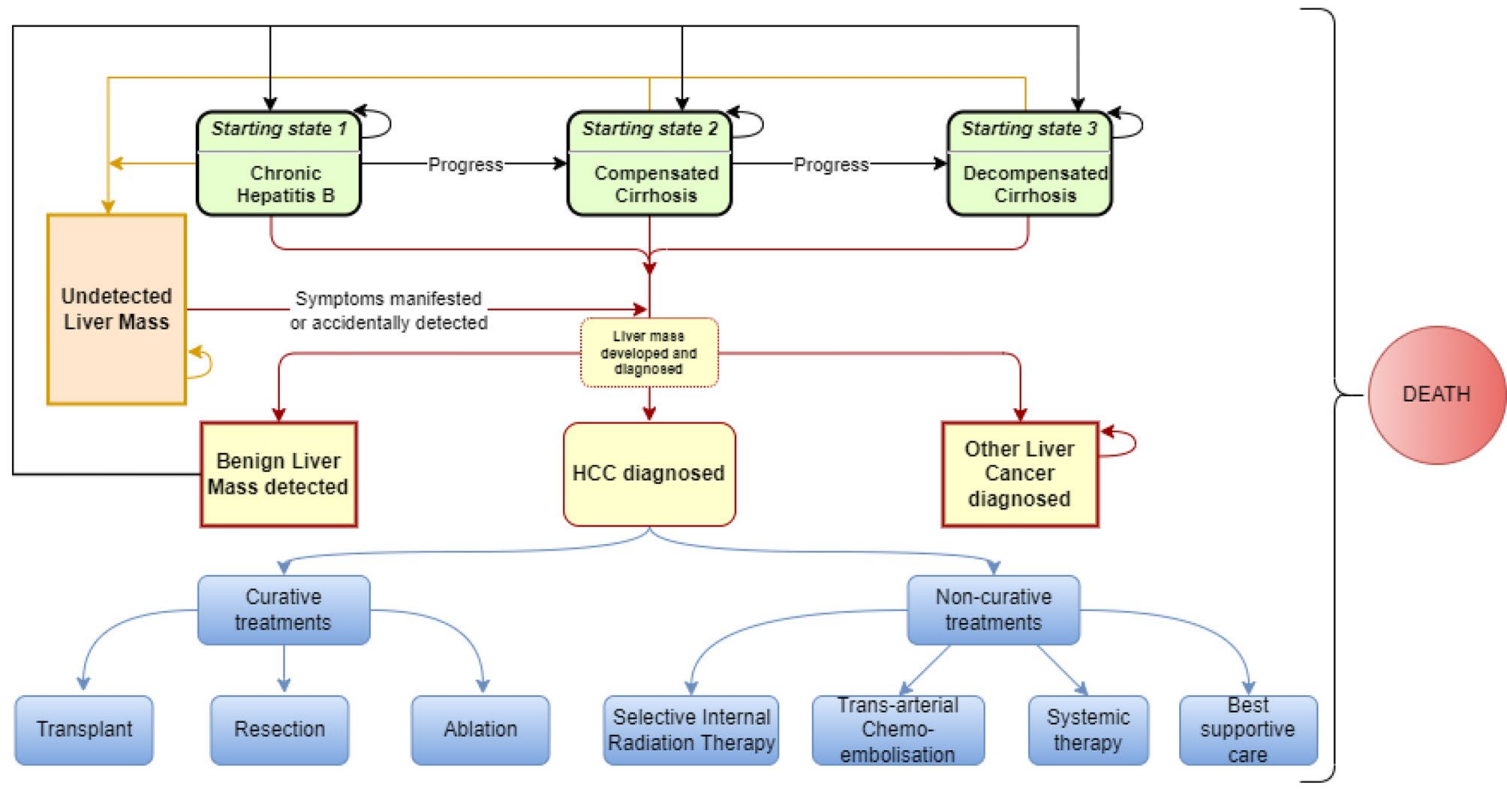
Structure of the state-transition individual-level model

A state-transition individual-level (microsimulation) model was used to model the disease progression through the movement of multiple health states (Fig. 1) over a lifetime horizon. The individual’s characteristics (starting age of entering the model and treatment of CHB) and tracker variables for storing disease progression history of individuals were incorporated in the model. Transitions between health states occurred in 6-month cycles to reflect the biannual interval of surveillance strategies. Analyses were done using TreeAge Pro Health Care 2022 R1.2 (TreeAge Software, Williamstown, Massachusetts).

### Population of interest and scenario analyses

Due to limited data, the model was unable to differentiate the ethnicities and gender of individuals. Therefore, a hypothetical baseline population consisting of 10,000 individuals at high risk of HCC was used, which included people with liver cirrhosis or non-cirrhotic CHB.

Previous Australian research reported that 15% of patients did not have liver cirrhosis prior to HCC diagnosis [15], therefore the model’s baseline cohort was assumed to include 15% of CHB individuals without liver cirrhosis. The remaining cohort consisted of 10% with decompensated cirrhosis and 75% with compensated cirrhosis, with the ratio of 10:75 or 0.133 between the two liver diseases. This closely matched the ratio derived from the global burden of disease study in 2017, which estimated the prevalence of decompensated and compensated cirrhosis in Australia to be 76.3 and 553.4 per 100,000 people, respectively (76.3:553.4 = 0.137) [24]. To account for uncertainties, different scenario and threshold analyses were conducted, including:

- exclusively surveilling non-cirrhotic CHB, compensated cirrhosis or decompensated cirrhosis populations and determining the threshold of disease progression rates that result in non cost-effective surveillance strategies becoming cost-effective;
- adjusting the sensitivity of ultrasound and prevalence of obesity in Australia to account for the impact of central adiposity on the precision of surveillance strategies. The early detection rate (proportion of detecting BCLC stage 0/A HCC) of ultrasound was reduced from 0.491 [15] to 0.210 [25], representing a reduction of 42.8%. Due to the lack of data for the sensitivity of ultrasound + AFP on people with obesity, the early detection rate of ultrasound + AFP was assumed to be reduced by the same rate (42.8%), from 0.618 [26] to 0.264. The differences in early detection rates between obese and non-obese individuals was divided by three and added to the probabilities of HCC being categorised as the three remaining BCLC stages (B to D). The prevalence of obesity in Australia was used to categorise the characteristics of individuals. It was estimated that 27.9% of Australians aged 18 years and older were obese [27];
- varying starting ages of the cohort to 12 different ranges: 20–80 years, 30–80, 40–80, 50–80, 20–70, 30–70, 40–70, 50–70, 20–60, 30–60, 40–60, and 50–60. The distribution of age for Australian population was presented in Appendix B. This followed the Australian recommendations that surveillance should be carried out for individuals with liver cirrhosis regardless of their age, and sub-Saharan African born people age 20 years and older [21]. These analyses were run separately with hypothetical cohorts of 10,000 individuals.

### Model parameters and data sources

#### Transition probabilities

The transition probabilities used in the model are summarised in Appendix A. Data was obtained from the following sources in decreasing order of priority: studies conducted in Australia or meta-analysis studies, studies in countries with similar population characteristics (the USA or the UK), studies in other countries, and expert opinion. The 6-month transition probabilities for health states were obtained and derived from published studies and the background mortality was obtained from the Australian Bureau of Statistics life Tables [28, 29].

#### Costs and effectiveness measured

Costs were reported from the health system perspective and only direct medical costs were included (Table A2, Appendix A). Costs were reported in 2019 Australian Dollar and inflated using the total health price index and the Government final consumption expenditure on hospitals and nursing homes (Table A3, Appendix A) [30]. Costs of surveilling (ultrasound and AFP) and diagnostic tests (MRI, CT, and biopsy) were obtained from the Medicare Benefit Schedule (MBS) [31]. All HCC treatment costs were sourced from the MBS except for liver transplant [32], liver resection [33], systemic therapy [34, 35] and best supportive care [36].

Health state utility values (HSUVs) were used to calculate Quality Adjusted Life Years (QALYs – outcome of effectiveness considered in this model) and obtained from different published studies (Table A2, Appendix A). HSUVs for CHB were derived by subtracting the Australian population norms for specific age groups [37] by disability weight for CHB people (Table 5A.2) [38]. HSUV for compensated cirrhosis was obtained from Australian paper using Short Form 36 questionnaire [39]. For decompensated cirrhosis, HSUV was obtained from another health economic modelling study [40], which weighted the average HSUV based on the number of respondents in each country who participated in a multi-national study conducted by Levy et al. using the standard gamble technique [41]. The HSUVs after HCC treatments were obtained from other modelling studies due to lack of published studies for these values. For systemic therapy, the HSUV was obtained by subtracting 1 by the 2019 Global Burden of Disease Study disability weight for sequela *“Terminal phase of liver cancer with medication”* [42].

Both costs and effectiveness were discounted by 5%, which was in line with the Australian guideline [43].

### Assumptions

Several assumptions were made in this study due to unavailability of data and model simplicity:

- Due to the lack of data for migrant groups at different ages, CHB individuals at different age groups and ethnicities being recommended for surveillance were categorised as the non-cirrhotic CHB group in the model. The risks of developing compensated cirrhosis and HCC were assumed to be the same for all individuals within this group and only differed by antiviral treatment for CHB.
- In the surveillance group, liver masses were identified by the surveillance strategy and then confirmed and characterised by either computed tomograpy (CT) or magnetic resonance imaging (MRI) scans. Meanwhile, in the non-surveillance groups, HCC was only detected when it became symptomatic. For tumour diagnosis, CT was assumed by expert opinion to be used in 90% of the total cases, and MRI in the remaining 10%. Indeterminate results were assumed to occur in 10% of cases; therefore, liver biopsy was assumed to be conducted for diagnosis.
- Adherence to surveillance was the same for both ultrasound and ultasound + AFP strategies.
- All treatment options took place within the same 6-month cycle as HCC diagnosis. Only one primary treatment was assumed for each cycle: after each cycle, the individual may have undergone different treatments or no treatments at all. Only those who underwent curative treatment options (liver transplant, resection, and ablation) had the risk of HCC recurrence. Recurrence was intrahepatic as only HCC treatments were modelled.
- The model stopped accumulating costs and effectiveness of individuals who were diagnosed with other types of liver cancer. Other types of liver cancer were assumed to be cholangiocarcinoma, the second most common type of liver cancer.
- All malignant liver masses smaller than 10 mm in diameter detected by ultrasound ± AFP became larger than 10 mm at the next cycle (after 6 months). Benign liver tumours were assumed to not progress to becoming malignant and required no treatment.

### Analysis

The main outcome of interest was the incremental cost-effectiveness ratio (ICER), which was calculated using the following formula:

$$ICER = \frac{Cost \left(strategy A\right)-Cost \left(strategy B\right)}{QALY \left(strategy A\right)-QALY \left(strategy B\right)}$$

This is interpreted as the incremental costs incurred by surveillance strategies (strategy A) in order to gain an additional QALY in comparison with that of the *status quo* (strategy B). The ICER was then compared with the willingness to pay (WTP) threshold of AUD50,000/QALY gained to determine the cost-effectiveness of surveillance strategies [44].

One-way sensitivity analyses were conducted on all transition probabilities, costs, and HSUVs to identify the most influential parameters on the ICERs. The range for sensitivity analyses of input parameters are included in Appendix A. The 20 most influential parameters are presented in the form of tornado diagrams.

The probabilistic sensitivity analyses were also undertaken to investigate multiple parameter uncertainties simultaneously. The Monte Carlo simulation was run 10,000 times with input values randomly drawn from relevant distributions to produce cost-effectiveness acceptability curves and incremental cost-effectiveness scatter plots for surveillance strategies against the *status quo*. The gamma and triangular distributions were assigned to costs and treatments for HCC at different BCLC stages, respectively, whilst the beta distribution was assigned to the remaining input parameters.

## Results

The costs and QALYs of 60 HCC surveillance scenarios (i.e., two HCC surveillance strategies with real-world and full adherence to surveillance compared to the *status quo* across 12 different ranges of cohort starting age) are shown in Table 1 for the baseline population. Overall, surveillance at biannual intervals using ultrasound with AFP was the most cost-effective at all ages. Ultrasound + AFP surveillance with a 100% adherence rate resulted in the highest rate of HCC diagnosed at an early stage, along with the highest QALYs and costs. This generated an ICER below $40,000/QALY gained compared to the *status quo* at all age ranges, which were all considered cost-effective when the WTP threshold of $50,000/QALY gained was adopted. Ultrasound + AFP surveillance at real-world adherence rates was also cost-effective with an ICERs of below $35,000/QALY gained against the *status quo*. Ultrasound surveillance alone had ICERs well below $50,000/QALY gained compared to the *status quo* but was extendedly dominated when ultrasound was combined with AFP.

**Table 1:** Cost-effectiveness analysis results of HCC surveillance: baseline population

Cohort starting age	Surveillance strategy	% Of early-stage HCC diagnosed	Cost	Incr. cost vs. next most cost-effective alternative	QALY	Incr. QALY vs. next most cost-effective alternative	ICER vs. next most cost-effective alternative	ICER vs. *status quo*	Dominance
20–80	*Status quo*	8.7	56,884	-	5.55	-	-	-	
	Ultrasound_real-world adherence	21.9	57,964	1,080	5.59	0.04	30,247	30,247	Extendedly dominated^†^
	Ultrasound + AFP_real-world adherence	25.8	58,557	1,673	5.61	0.06	29,174	29,174	
	Ultrasound_full adherence	48.6	59,390	833	5.62	0.01	124,852	39,146	Extendedly dominated
	Ultrasound + AFP_full adherence	61.9	60,435	1,878	5.66	0.05	38,362	33,406	
30–80	*Status quo*	8.4	55,992		5.42				
	Ultrasound_real-world adherence	22.2	57,104	1,112	5.46	0.04	31,137	31,137	Extendedly dominated
	Ultrasound + AFP_real-world adherence	26.1	57,727	1,735	5.48	0.06	30,174	30,174	
	Ultrasound_full adherence	49.0	58,597	870	5.49	0.01	84,099	38,399	Extendedly dominated
	Ultrasound + AFP_full adherence	61.8	59,672	1,945	5.53	0.05	37,028	33,446	
40–80	*Status quo*	8.6	54,986		5.25				
	Ultrasound_real-world adherence	22.9	56,029	1,042	5.28	0.03	32,668	32,668	Extendedly dominated
	Ultrasound + AFP_real-world adherence	26.7	56,581	1,594	5.30	0.05	30,967	30,967	
	Ultrasound_full adherence	49.4	57,348	768	5.31	0.01	134,702	41,305	Extendedly dominated
	Ultrasound + AFP_full adherence	62.0	58,276	1,696	5.34	0.04	40,466	35,229	
50–80	*Status quo*	8.1	53,215		5.04				
	Ultrasound_real-world adherence	22.8	54,224	1,009	5.07	0.03	37,429	37,429	Extendedly dominated
	Ultrasound + AFP_real-world adherence	26.8	54,787	1,572	5.09	0.05	34,363	34,363	
	Ultrasound_full adherence	49.4	55,401	614	5.09	0.01	104,021	42,320	Extendedly dominated
	Ultrasound + AFP_full adherence	61.8	56,251	1,464	5.13	0.04	38,521	36,250	
20–70	*Status quo*	8.3	58,090		5.66				
	Ultrasound_real-world adherence	21.5	59,179	1,089	5.70	0.04	29,702	29,702	Extendedly dominated
	Ultrasound + AFP_real-world adherence	25.1	59,813	1,722	5.72	0.06	29,126	29,126	
	Ultrasound_full adherence	48.9	60,682	869	5.73	0.01	86,527	37,461	Extendedly dominated
	Ultrasound + AFP_full adherence	61.9	61,757	1,945	5.78	0.05	36,228	32,505	
30–70	*Status quo*	8.7	57,531		5.59				
	Ultrasound_real-world adherence	22.1	58,602	1,070	5.62	0.03	31,002	31,002	Extendedly dominated
	Ultrasound + AFP_real-world adherence	25.8	59,206	1,675	5.64	0.06	29,740	29,740	
	Ultrasound_full adherence	48.8	60,136	930	5.65	0.01	110,574	40,241	Extendedly dominated
	Ultrasound + AFP_full adherence	62.1	61,162	1,956	5.69	0.05	38,243	33,787	
40–70	*Status quo*	8.7	56,774		5.45				
	Ultrasound_real-world adherence	22.2	57,823	1,049	5.48	0.03	34,367	34,367	Extendedly dominated
	Ultrasound + AFP_real-world adherence	26.0	58,411	1,637	5.50	0.05	31,440	31,440	
	Ultrasound_full adherence	48.7	59,204	793	5.51	0.01	86,237	39,660	Extendedly dominated
	Ultrasound + AFP_full adherence	61.7	60,209	1,798	5.55	0.05	36,098	33,717	
50–70	*Status quo*	8.3	55,562		5.28				
	Ultrasound_real-world adherence	22.4	56,551	989	5.31	0.03	34,371	34,371	Extendedly dominated
	Ultrasound + AFP_real-world adherence	26.2	57,161	1,600	5.33	0.05	31,757	31,757	
	Ultrasound_full adherence	49.2	57,895	733	5.34	0.01	114,832	41,105	Extendedly dominated
	Ultrasound + AFP_full adherence	62.0	58,864	1,703	5.38	0.05	37,549	34,501	
20–60	*Status quo*	8.5	59,044		5.80				
	Ultrasound_real-world adherence	21.4	60,132	1,089	5.83	0.03	31,747	31,747	Extendedly dominated
	Ultrasound + AFP_real-world adherence	25.0	60,750	1,707	5.85	0.06	29,948	29,948	
	Ultrasound_full adherence	48.9	61,633	883	5.86	0.01	86,367	38,530	Extendedly dominated
	Ultrasound + AFP_full adherence	61.9	62,730	1,979	5.91	0.06	35,220	32,565	
30–60	*Status quo*	8.4	58,560		5.72				
	Ultrasound_real-world adherence	21.6	59,646	1,086	5.75	0.03	31,490	31,490	Extendedly dominated
	Ultrasound + AFP_real-world adherence	25.3	60,252	1,691	5.78	0.06	30,038	30,038	
	Ultrasound_full adherence	49.0	61,117	865	5.79	0.01	69,755	37,207	Extendedly dominated
	Ultrasound + AFP_full adherence	62.0	62,186	1,934	5.83	0.06	33,993	32,026	
40–60	*Status quo*	8.4	58,002		5.61				
	Ultrasound_real-world adherence	21.7	59,065	1,063	5.65	0.03	32,191	32,191	Extendedly dominated
	Ultrasound + AFP_real-world adherence	25.5	59,666	1,664	5.67	0.05	30,412	30,412	
	Ultrasound_full adherence	49.0	60,563	897	5.68	0.01	66,743	37,572	Extendedly dominated
	Ultrasound + AFP_full adherence	62.0	61,608	1,941	5.72	0.06	35,159	32,796	
50–60	*Status quo*	8.5	56,951		5.47				
	Ultrasound_real-world adherence	22.2	58,018	1,067	5.50	0.03	31,356	31,356	Extendedly dominated
	Ultrasound + AFP_real-world adherence	26.1	58,621	1,670	5.52	0.06	30,008	30,008	
	Ultrasound_full adherence	48.8	59,460	839	5.53	0.01	95,148	38,915	Extendedly dominated
	Ultrasound + AFP_full adherence	61.8	60,483	1,862	5.57	0.05	37,770	33,653	

*AFP; Alpha-Fetoprotein; HCC, hepatocellular carcinoma; ICER, Incremental cost-effectiveness ratio; Incr., incremental; QALY, quality adjusted life years*

† *A strategy with a higher ICER (relative to the next alternative) and lower QALY than the alternative was extendedly dominated by the alternative*

Cost-effectiveness results for exclusive surveillance for people with non-cirrhotic CHB or compensated cirrhosis or decompensated cirrhosis alone are shown in Appendix C, Table C1-C3, with 60 surveillance strategies for each of the three separate hypothetical cohorts. Surveillance was cost-effective in the compensated or decompensated cirrhosis populations alone (ICERs < $30,000/QALY gained against *status quo*), but not cost-effective in the CHB population (ICERs > $100,000/QALY gained against *status quo*). Furthermore, surveillance with a 100% adherence rate was more cost-effective than surveillance with real-world adherence rates for compensated and decompensated cirrhosis populations. Threshold analyses were then conducted to determine the threshold of disease progression rate at which HCC surveillance in the CHB population became cost-effective (Figure D1, D2, Appendix D). The transition probabilities for CHB individuals not undergoing antiviral treatment to compensated cirrhosis (0.0075) and liver mass (0.0013) needed to increase to above 0.0650 and 0.0050 respectively to make surveillance in CHB individuals cost-effective. The transition probabilities of individuals undergoing CHB treatments was not considered in the threshold analysis due to its minimal impact on ICER.

The costs and QALYs of 60 HCC surveillance strategies when the impact of central adiposity on ultrasound being considered is shown in Table 2 for the baseline population. The rate of early-stage HCC being diagnosed decreased whilst the ICER of surveillance strategies compared to the *status quo* increased substantially. Nevertheless, all ICERs falling below the WTP threshold meant surveillance using ultrasound + AFP was still cost-effective if it was conducted in a population with up to 27.9% of obese individuals.

**Table 2:** Cost-effectiveness analysis results of HCC surveillance: central adiposity

Cohort starting age	Surveillance strategy	% Of early-stage HCC diagnosed	Cost	Incr. cost vs. next most cost-effective alternative	QALY	Incr. QALY vs. next most cost-effective alternative	ICER vs. next most cost-effective alternative	ICER vs. *status quo*	Dominance
20–80	*Status quo*	8.7	56,884		5.55				
	Ultrasound_real-world adherence	19.5	57,879	995	5.58	0.02	40,222	40,222	Extendedly dominated^†^
	Ultrasound + AFP_real-world adherence	22.9	58,408	1,524	5.60	0.04	34,249	34,249	
	Ultrasound_full adherence	41.5	59,234	826	5.59	-0.01	-99,541	64,894	Absolutely dominated^§^
	Ultrasound + AFP_full adherence	53.2	60,279	1,871	5.63	0.03	59,522	44,710	
30–80	*Status quo*	8.4	55,992		5.42				
	Ultrasound_real-world adherence	19.8	57,037	1,045	5.45	0.03	41,003	41,003	Extendedly dominated
	Ultrasound + AFP_real-world adherence	23.2	57,598	1,606	5.47	0.05	35,271	35,271	
	Ultrasound_full adherence	41.8	58,467	869	5.46	-0.00	-201,771	60,033	Absolutely dominated
	Ultrasound + AFP_full adherence	52.8	59,549	1,951	5.50	0.03	56,166	44,311	
40–80	*Status quo*	8.6	54,986		5.25				
	Ultrasound_real-world adherence	20.3	55,972	986	5.27	0.02	42,545	42,545	Extendedly dominated
	Ultrasound + AFP_real-world adherence	23.7	56,445	1,459	5.29	0.04	36,258	36,258	
	Ultrasound_full adherence	42.1	57,236	791	5.28	-0.01	-87,612	72,074	Absolutely dominated
	Ultrasound + AFP_full adherence	53.1	58,159	1,714	5.32	0.02	71,394	49,387	
50–80	*Status quo*	8.1	53,215		5.04				
	Ultrasound_real-world adherence	20.3	54,162	947	5.06	0.02	54,071	54,071	Extendedly dominated
	Ultrasound + AFP_real-world adherence	23.7	54,663	1,448	5.08	0.03	41,830	41,830	
	Ultrasound_full adherence	42.1	55,318	655	5.07	-0.01	-97,534	75,385	Absolutely dominated
	Ultrasound + AFP_full adherence	52.8	56,227	1,564	5.10	0.03	61,866	50,287	
20–70	*Status quo*	8.3	58,090		5.66				
	Ultrasound_real-world adherence	19.1	59,093	1,003	5.69	0.03	39,210	39,210	Extendedly dominated
	Ultrasound + AFP_real-world adherence	22.3	59,665	1,574	5.71	0.05	34,007	34,007	
	Ultrasound_full adherence	41.7	60,492	828	5.70	-0.01	-105,045	62,528	Absolutely dominated
	Ultrasound + AFP_full adherence	52.9	61,614	1,949	5.75	0.03	56,870	43,733	
30–70	*Status quo*	8.7	57,531		5.59				
	Ultrasound_real-world adherence	19.7	58,518	987	5.61	0.02	41,068	41,068	Extendedly dominated
	Ultrasound + AFP_real-world adherence	23.0	59,060	1,529	5.63	0.04	34,816	34,816	
	Ultrasound_full adherence	41.6	59,940	879	5.62	-0.01	-107,583	67,387	Absolutely dominated
	Ultrasound + AFP_full adherence	52.8	61,014	1,954	5.66	0.03	60,301	45,636	
40–70	*Status quo*	8.7	56,774		5.45				
	Ultrasound_real-world adherence	19.8	57,738	964	5.47	0.02	48,459	48,459	Extendedly dominated
	Ultrasound + AFP_real-world adherence	23.2	58,266	1,492	5.49	0.04	37,674	37,674	
	Ultrasound_full adherence	41.6	59,048	782	5.48	-0.01	-123,501	68,343	Absolutely dominated
	Ultrasound + AFP_full adherence	52.9	60,078	1,812	5.52	0.03	54,921	45,513	
50–70	*Status quo*	8.3	55,562		5.28				
	Ultrasound_real-world adherence	19.9	56,481	919	5.30	0.02	47,382	47,382	Extendedly dominated
	Ultrasound + AFP_real-world adherence	23.3	57,026	1,464	5.32	0.04	37,316	37,316	
	Ultrasound_full adherence	41.8	57,730	704	5.31	-0.01	-72,520	73,459	Absolutely dominated
	Ultrasound + AFP_full adherence	53.2	58,734	1,708	5.35	0.03	61,182	47,239	
20–60	*Status quo*	8.5	59,044		5.80				
	Ultrasound_real-world adherence	19.2	60,056	1,012	5.82	0.02	42,757	42,757	Extendedly dominated
	Ultrasound + AFP_real-world adherence	22.4	60,607	1,564	5.84	0.04	35,136	35,136	
	Ultrasound_full adherence	41.8	61,439	832	5.83	-0.01	-101,613	65,954	Absolutely dominated
	Ultrasound + AFP_full adherence	53.0	62,575	1,968	5.88	0.03	56,373	44,470	
30–60	*Status quo*	8.4	58,560		5.72				
	Ultrasound_real-world adherence	19.3	59,558	998	5.74	0.02	42,470	42,470	Extendedly dominated
	Ultrasound + AFP_real-world adherence	22.5	60,104	1,544	5.76	0.04	35,356	35,356	
	Ultrasound_full adherence	41.8	60,930	826	5.76	-0.00	-171,248	61,009	Absolutely dominated
	Ultrasound + AFP_full adherence	53.0	62,037	1,933	5.80	0.04	51,496	42,816	
40–60	*Status quo*	8.4	58,002		5.61				
	Ultrasound_real-world adherence	19.4	58,973	971	5.64	0.02	44,327	44,327	Extendedly dominated
	Ultrasound + AFP_real-world adherence	22.7	59,518	1,516	5.66	0.04	36,228	36,228	
	Ultrasound_full adherence	41.8	60,371	853	5.65	-0.00	-175,380	64,048	Absolutely dominated
	Ultrasound + AFP_full adherence	53.1	61,450	1,932	5.69	0.03	55,314	44,910	
50–60	*Status quo*	8.5	56,951		5.47				
	Ultrasound_real-world adherence	19.8	57,938	987	5.49	0.02	42,155	42,155	Extendedly dominated
	Ultrasound + AFP_real-world adherence	23.2	58,480	1,529	5.51	0.04	35,170	35,170	
	Ultrasound_full adherence	41.6	59,307	827	5.50	-0.01	-100,712	66,787	Absolutely dominated
	Ultrasound + AFP_full adherence	53.1	60,343	1,863	5.54	0.03	61,130	45,865	

*AFP; Alpha-Fetoprotein; HCC, hepatocellular carcinoma; ICER, Incremental cost-effectiveness ratio; Incr., incremental; QALY, quality adjusted life years*

† *A strategy with a higher ICER (relative to the next alternative) and lower QALY than the alternative was extendedly dominated by the alternative*

§ *A strategy with higher cost and lower QALY than the alternative was absolutely dominated by the alternative*

**Fig. 2:**
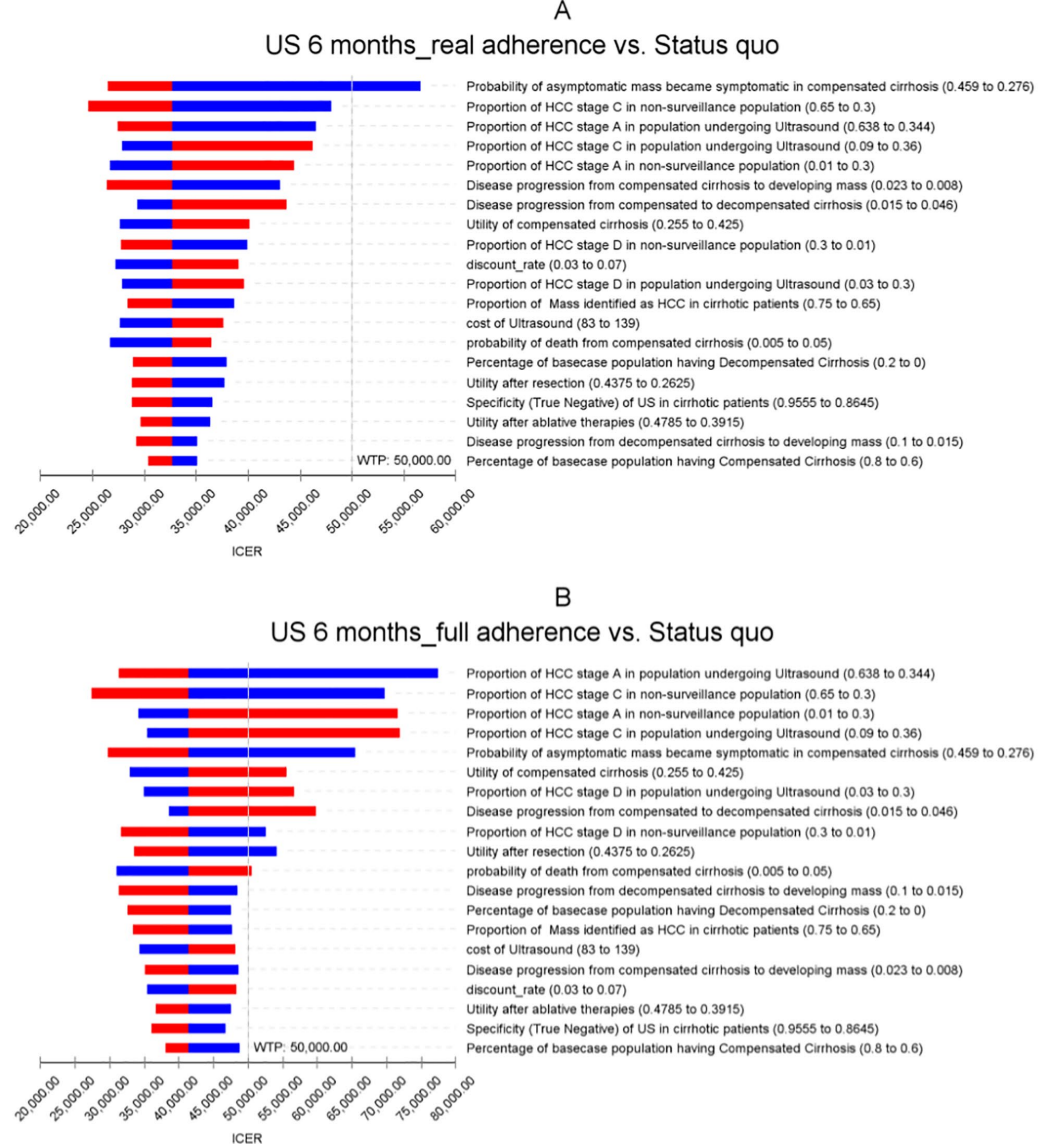
Tornado diagram of Ultrasound surveillance on baseline population at age range 40–80

**Fig. 3:**
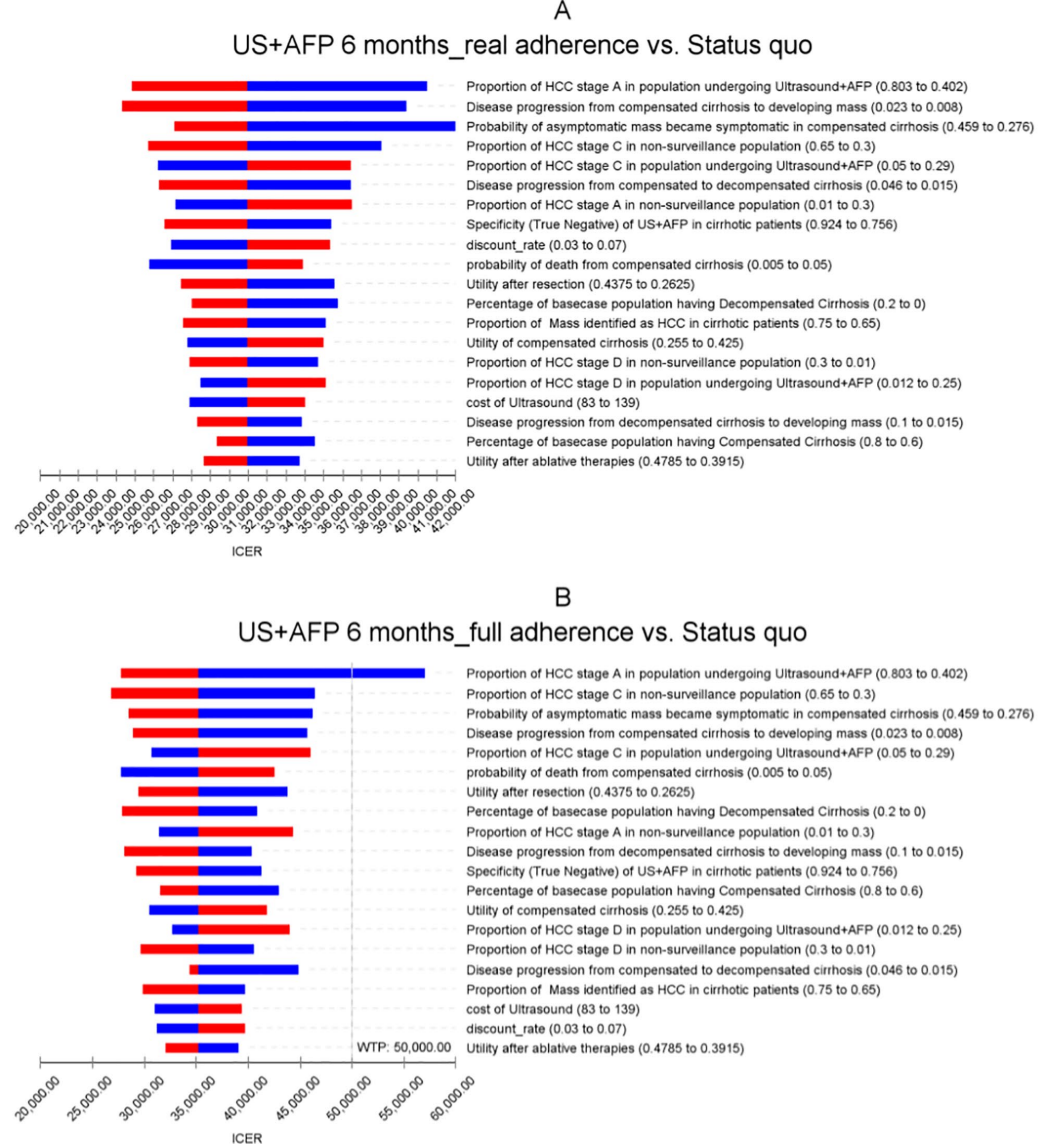
Tornado diagram of Ultrasound + AFP surveillance on baseline population at age range 40–80

Results from one-way sensitivity analyses were expressed in the form of tornado diagrams in Figs. 2 and 3. The baseline population with starting age range of 40 to 80 years was chosen to report the sensitivity analyses results as using other ranges of age only produced small changes in the results. Other ranges of age were not reported due to minimal differences in the results. The most influential parameters on the ICER of biannual ultrasound at real-world adherence rates against the *status quo* were the probability of an asymptomatic mass became symptomatic in compensated cirrhosis, the proportion of HCC stage C in non-surveillance populations, and proportion of HCC stage A in populations undergoing surveillance. For surveillance using ultrasound + AFP at real-world adherence rates, the most influential parameters were the proportion of HCC stage A in populations undergoing ultrasound + AFP, disease progression from compensated cirrhosis to developing liver masses, and the probability of an asymptomatic mass becoming symptomatic in compensated cirrhosis.

**Fig. 4:**
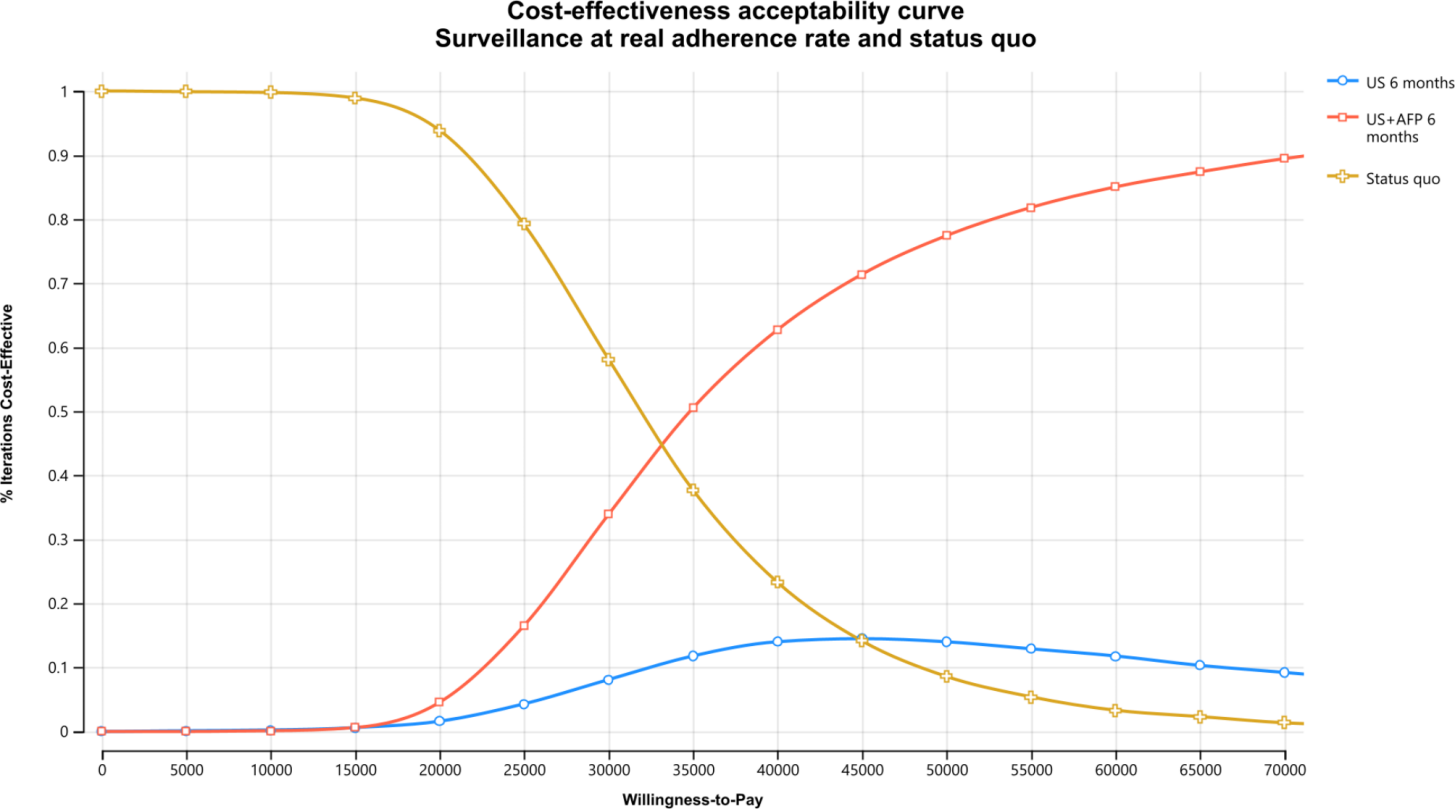
Cost-effectiveness acceptability curves for surveillance at real adherence rate and *status quo* using baseline population aged 40 to 80 years

Results from 10,000 Monte Carlo simulations for probabilistic sensitivity analyses are illustrated as cost-effectiveness acceptability curve in Fig. 4 and incremental cost-effectiveness scatter plots in Figures D3 and D4, Appendix D. The *status quo* had the highest probability of being cost-effective if the WTP threshold was below $33,000/QALY gained. If the threshold was set at $50,000/QALY gained, ultrasound + AFP surveillance was cost-effective in 77.5% of the simulations, whilst the cost-effectiveness probabilities for ultrasound surveillance and the *status quo* were 13.4% and 8.5%, respectively.

## Discussion

The results from our model showed HCC surveillance based on Australian recommendations using biannual ultrasound with or without AFP was cost-effective in comparison with the *status quo* or no formal surveillance. However, combining AFP with recurring ultrasound was more cost-effective than ultrasound alone due to the lower ICER.

The adherence rate to HCC surveillance has only been taken into account in a small number of economic evaluations of HCC surveillance in the past and those studies revealed higher adherence rates were associated with higher costs and effectiveness of the surveillance [4]. Our model showed surveillance with real-world adherence rates was extendedly dominated by a fully adhered surveillance program for compensated and decompensated cirrhosis populations. It is worth noting that surveillance with a 100% adherence rate is infeasible to achieve in reality, even for population-based programs in Australia such as breast cancer surveillance. Only 60.9% of Australian women aged 50 to 72 years were reported to return for their next breast cancer surveillance round in 2017 [45]. Nevertheless, even though failure to adhere to regular surveillance might reduce the cost-effectiveness of surveillance [46, 47], our model showed HCC surveillance was still cost-effective when it incorporated the real-world adherence rate.

Given the complexity of economic models, certain levels of uncertainty always exist around the parameters, characteristics of population, process of microsimulation, and structure of the model itself [48], especially when the model is run on a lifetime horizon. However, results from our model validation (Appendix E) have shown that the model’s outcomes were predominantly consistent with the real-world data. We also followed good research practice for model parameter estimation and uncertainty [48] by conducting multiple one-way and probabilistic sensitivity analyses together with scenario and threshold analyses. Whilst varying the model’s parameters had an impact on the costs, QALYs and resulting ICER for each strategy, the sensitivity analyses showed the decision as to whether or not the ultrasound + AFP surveillance were considered cost effective mostly did not change. Only a reduction in the proportion of HCC stage A in the population undergoing ultrasound + AFP surveillance would make the ICER for this surveillance approach (with full adherence rate) exceed the WTP threshold. It should also be pointed out that most of the model’s parameters were varied at a relatively wide range for one-way sensitivity analyses.

Uncertainties around the baseline hypothetical cohort used in our model were thoroughly investigated by addressing exclusive surveillance of three separate cohorts and adjusting different ranges of starting age for the cohort. Furthermore, whilst the percentages of the base population having compensated and decompensated cirrhosis were shown to have an impact on ICERs in the tornado diagram, varying those percentages by a large extent did not change the conclusion that HCC surveillance was cost-effective. With the Australian recommendation that HCC surveillance should be offered to all patients with cirrhosis regardless of age, our model showed all the strategies for cirrhotic patients were cost-effective over different ranges of starting age. Our findings were in line with another health economic study conducted in Australia, showing 6-month ultrasound surveillance had an ICER of $23,090/QALY gained versus the *status quo* [49]. We also found exclusively surveilling CHB people was not cost-effective due to the low progression rate from CHB to compensated cirrhosis or HCC, which resulted in a minor gain of QALYs compared to the *status quo*. Our findings were comparable to previous economic evaluations on HCC surveillance for CHB in Australia in 2009, showing biannual ultrasound + AFP had an unfavourable ICER (> $400,000/QALY gained) against the current practice [50]. Nevertheless, compared to these previous works, we assessed a wide variety of starting age ranges for the cohort and took into account real-world adherence rate to reinforce our evaluation results. Our threshold analysis showed the progression rate of CHB would need to increase several folds in order for surveillance to be cost-effective in this population.

We also conducted several scenario analyses to address the impact of obesity/central adiposity on the sensitivity of ultrasound. The results showed central adiposity could reduce the cost-effectiveness of ultrasound ± AFP due to the lower rate of HCC being diagnosed at early stages, but the strategies remained cost-effective. However, due to the lack of data for sensitivity of ultrasound + AFP on people with obesity/central adiposity, the early detection rate of this strategy was derived from data on ultrasound surveillance for people with obesity. Future studies could investigate the diagnostic performance of ultrasound + AFP surveillance on people with obesity so that more robust data could be inputted to our model.

Whilst the model was built to closely reflect the Australian recommendations for HCC surveillance and management, structural and input parameters of the model can be modified to conduct cost-effectiveness analyses in other healthcare settings. Even though our model demonstrates the cost-effectiveness of HCC surveillance in Australia, it still has several limitations. Due to the lack of Australian data for several input parameters of our model, we relied on studies published for other countries. As these studies may not truly reflect the disease status in Australia, we tried to mitigate this risk by prioritising data from countries with similar population and clinical characteristics as Australia. Furthermore, the risks of developing compensated cirrhosis and HCC were assumed to be the same for all individuals with CHB and constant over time, whilst there are possible variations of risks amongst different age groups, ethnicities, and gender of the CHB population. It is also due to this assumption that the model was unable to simultaneously simulate all the diverse CHB populations recommended for surveillance: Asian men older than 40 years, Asian women older than 50, Sub-Saharan African people older than 20, and Aboriginal and Torres Strait Islander people older than 50. Instead, these groups were included in the non-cirrhotic CHB group. This limitation was addressed by conducting different scenarios and threshold analyses, but certain level of uncertainties might still remain. Future robust studies on age-dependent disease progression of CHB for people of culturally diverse backgrounds would provide important inputs for this model to improve its outcomes. Another limitation was that the HSUVs for treatment after the diagnosis of HCC were mostly obtained from other economic modelling studies and assumptions, which might not accurately represent the utilities of this Australian population.

Considering the likely cost-effectiveness of HCC surveillance, decisions can be made in regard to resource allocation for surveillance programs in Australia at a larger and systematic scale. Efforts are also needed to increase awareness of HCC surveillance amongst healthcare providers and patients, and to address any barriers to access or adherence to surveillance. This may involve the establishment of targeted education and awareness campaigns, the provision of adequate resources and personnel, and the implementation of policies and reimbursement models that support HCC surveillance.

## Conclusions

HCC surveillance based on Australian recommendations using biannual ultrasound with or without AFP was cost-effective. However, combining ultrasound with AFP was more cost-effective than ultrasound alone due to its lower ICER. Sub-group analyses showed surveillance limited to people with cirrhosis was cost-effective, but for only CHB people, surveillance would exceed the cost-effectiveness threshold. The impact of obesity increased the ICER of surveillance compared to the *status quo*, but the results were within the accepted WTP threshold.

## Electronic supplementary material

Below is the link to the electronic supplementary material.

Appendix A. Input paratemeters of the model. Table A1. Transition probabilities at 6-month interval, proportion values and discount rate used in the model. Table A2. Costs values (inflated to 2019-20 price)* at 6-month interval and Health state utility values (HSUV) used in the model. Table A3. Health price index, 2013/14 to 2019/20 (reference year 2019/20: 100) [30]. Table A5. Treatments for HCC at different BCLC stages (Triangular distribution for PSA) [32]. Table A6. Cumulative mortality rate of Cholangiocarcinoma at time of diagnosis [33]. Table A7. Cumulative mortality rate after liver transplant [12]. Table A8. Annual recurrence rate after liver resection [34]. Table A9. Cumulative recurrence rate after liver ablation [35]. Table A10. Treatments after HCC recurrence (Beta distribution for PSA with Standard deviation = 20% of the mean.Appendix B. Distribution of age at baseline in Australia, 2020. Table B1. Number and percentage of Australians by age. Figure B1. Distribution of age at baseline. Appendix C. Cost-effectiveness results of exclusive surveillance for non-cirrhotic CHB, compensated cirrhosis and decompensated cirrhosis population. Table C1. Cost-effectiveness of surveillance strategies on non-cirrhotic CHB people only. Table C2. Cost-effectiveness of surveillance strategies on compensated cirrhosis patients only.Table C3. Cost-effectiveness of surveillance strategies on decompensated cirrhosis patients only. Appendix D. Results of sensitivity and threshold analyses. Figure D2. Disease progression rates from CHB to compensated cirrhosis at different age ranges: (A) 20 to 80; (B) 30 to 80; (C) 40 to 80; (D) 50 to 80. Figure D3. Incremental cost-effectiveness scatterplot for Ultrasound+AFP versus status quo at real-world adherence rates, baseline population aged 40 to 80 years (green points represent optimal strategies, eclipse area represents 95% confidence interval). Figure D4. Incremental cost-effectiveness scatterplot for Ultrasound versus status quo at real-world adherence rate, baseline population aged 40 to 80 years (green points represent optimal strategies, eclipse area represents 95% confidence interval). Appendix E. Validation of the model. Table E1. Internal validity results. Table E2. External validity results. Table E3. Mean error values of internal and external validation. Figure E. Goodness-of-fit results of the model. Appendix F. Consolidated Health Economic Evaluation Reporting Standards (CHEERS) Checklist. Appendix G. Reference

## Data Availability

Study materials are available from the corresponding author upon reasonable request.

## List of abbreviations

AFP: Alpha-fetoprotein
BCLC: Barcelona Clinic Liver Cancer
CHB: Chronic Hepatitis B
CT: Computed tomography
HCC: Hepatocellular carcinoma
HSUV: Health state utility value
ICER: Incremental cost-effectiveness ratio
MRI: Magnetic resonance imaging
PLC: Primary liver cancer
QALY: Quality-adjusted life year
WTP: Willingness to pay

## References

[1] Mortality GBD, Causes of Death C (2016). Global, regional, and national life expectancy, all–cause mortality, and cause–specific mortality for 249 causes of death, 1980–2015: a systematic analysis for the global burden of Disease Study 2015. Lancet.

[2] Yang JD, Hainaut P, Gores GJ, Amadou A, Plymoth A, Roberts LR (2019). A global view of hepatocellular carcinoma: trends, risk, prevention and management. Nat Rev Gastroenterol Hepatol.

[3] Cocker F, Chien Yee K, Palmer AJ, de Graaff B (2019). Increasing incidence and mortality related to liver cancer in Australia: time to turn the tide. Aust N Z J Public Health.

[4] Nguyen ALT, Nguyen HTT, Yee KC, Palmer AJ, Blizzard CL, de Graaff B (2021). A systematic review and narrative synthesis of Health economic evaluations of Hepatocellular Carcinoma screening strategies. Value Health.

[5] Patel N, Yopp AC, Singal AG (2015). Diagnostic delays are common among patients with hepatocellular carcinoma. J Natl Compr Canc Netw.

[6] Cabibbo G, Craxi A (2009). Hepatocellular cancer: optimal strategies for screening and surveillance. Dig Dis (Basel Switzerland).

[7] Balogh J, Victor D 3rd, Asham EH, Burroughs SG, Boktour M, Saharia A, Li X, Ghobrial RM, Monsour HP. Jr.: Hepatocellular carcinoma: a review. *J Hepatocell Carcinoma* 2016, 3:41–53. 10.2147/JHC.S61146 PMC5063561 27785449

[8] Thein HH, Isaranuwatchai W, Campitelli MA, Feld JJ, Yoshida E, Sherman M, Hoch JS, Peacock S, Krahn MD, Earle CC (2013). Health care costs associated with hepatocellular carcinoma: a population–based study. Hepatology.

[9] Tayob N, Lok ASF, Do KA, Feng ZD (2016). Improved detection of Hepatocellular Carcinoma by using a longitudinal alpha–fetoprotein screening algorithm. Clin Gastroenterol Hepatol.

[10] Pazgan–Simon M, Serafinska S, Janocha–Litwin J, Simon K, Zuwala–Jagiello J. Diagnostic Challenges in Primary Hepatocellular Carcinoma: Case Reports and Review of the Literature. Case Rep Oncol Med 2015. 10.1155/2015/878763 PMC4397422 25922775

[11] Liver Cancer Early Detection, Diagnosis and Staging [https://www.cancer.org/content/dam/CRC/PDF/Public/8700.00.pdf]

[12] Tsai WC, Kung PT, Wang YH, Kuo WY, Li YH. Influence of the time interval from diagnosis to treatment on survival for early–stage liver cancer. Plos One 2018, 13(6). 10.1371/journal.pone.0199532 PMC6014663 29933395

[13] Dimitroulis D, Damaskos C, Valsami S, Davakis S, Garmpis N, Spartalis E, Athanasiou A, Moris D, Sakellariou S, Kykalos S (2017). From diagnosis to treatment of hepatocellular carcinoma: an epidemic problem for both developed and developing world. World J Gastroentero.

[14] Ye SL, Takayama T, Geschwind J, Marrero JA, Bronowicki JP (2010). Current approaches to the treatment of early hepatocellular carcinoma. Oncologist.

[15] Hong TP, Gow PJ, Fink M, Dev A, Roberts SK, Nicoll A, Lubel JS, Kronborg I, Arachchi N, Ryan M (2018). Surveillance improves survival of patients with hepatocellular carcinoma: a prospective population–based study. Med J Australia.

[16] Khalaf N, Ying J, Mittal S, Temple S, Kanwal F, Davila J, El–Serag HB (2017). Natural history of untreated hepatocellular carcinoma in a US Cohort and the role of Cancer Surveillance. Clin Gastroenterol Hepatol.

[17] Hucke F, Sieghart W, Schoniger–Hekele M, Peck–Radosavljevic M, Muller C (2011). Clinical characteristics of patients with hepatocellular carcinoma in Austria – is there a need for a structured screening program?. Wien Klin Wochenschr.

[18] Heimbach JK, Kulik LM, Finn RS, Sirlin CB, Abecassis MM, Roberts LR, Zhu AX, Murad MH, Marrero JA (2018). AASLD guidelines for the treatment of hepatocellular carcinoma. Hepatology.

[19] European Organisation For L, Treatment Of R, European Association For The Study Of The (2012). EASL–EORTC clinical practice guidelines: management of hepatocellular carcinoma. J Hepatol.

[20] Omata M, Cheng AL, Kokudo N, Kudo M, Lee JM, Jia J, Tateishi R, Han KH, Chawla YK, Shiina S (2017). Asia–Pacific clinical practice guidelines on the management of hepatocellular carcinoma: a 2017 update. Hepatol Int.

[21] Lubel JS, Roberts SK, Strasser SI, Thompson AJ, Philip J, Goodwin M, Clarke S, Crawford DH, Levy MT, Shackel N (2021). Australian recommendations for the management of hepatocellular carcinoma: a consensus statement. Med J Aust.

[22] Pons F, Varela M, Llovet JM (2005). Staging systems in hepatocellular carcinoma. HPB (Oxford).

[23] Sarkar M, Stewart S, Yu A, Chen M, Nguyen TT, Khalili M (2012). Hepatocellular carcinoma screening practices and impact on survival among hepatitis B-infected asian Americans. J Viral Hepatitis.

[24] Collaborators GBDC (2020). The global, regional, and national burden of cirrhosis by cause in 195 countries and territories, 1990–2017: a systematic analysis for the global burden of Disease Study 2017. Lancet Gastroenterol Hepatol.

[25] Esfeh JM, Hajifathalian K, Ansari–Gilani K (2020). Sensitivity of ultrasound in detecting hepatocellular carcinoma in obese patients compared to explant pathology as the gold standard. Clin Mol Hepatol.

[26] van Meer S, Robert A, Coenraad MJ, Sprengers D, van Nieuwkerk KM, Klümpen HJ, Jansen PL, IJzermans JN, van Oijen MG, Siersema PD (2015). Surveillance for hepatocellular carcinoma is associated with increased survival: results from a large cohort in the Netherlands. J Hepatol.

[27] Sarin SK, Kumar M, Eslam M, George J, Al Mahtab M, Akbar SMF, Jia J, Tian Q, Aggarwal R, Muljono DH (2020). Liver diseases in the Asia–Pacific region: a Lancet Gastroenterology & Hepatology Commission. Lancet Gastroenterol Hepatol.

[28] Australian life tables reference period 2018–2020 [https://www.abs.gov.au/statistics/people/population/life-tables/latest-release]

[29] Regional population by age and sex reference period 2020 [https://www.abs.gov.au/statistics/people/population/regional-population-age-and-sex/latest-release]

[30] Health Expenditure Australia 2019–20: Data tables for Health Expenditure Australia 2019–20 [https://www.aihw.gov.au/reports/health-welfare-expenditure/health-expenditure-australia-2019-20/contents/summary]

[31] Australian Department of Health. : Medicare Benefits Schedule Book - Operating from 1 January 2020. In. Canberra ACT; 2020.

[32] The Independent Hospital Pricing Authority (IHPA). : National Hospital Cost Data Collection Report, Public Sector, Round 23 (Financial year 2018–19). In.; 2021.

[33] Cosic L, Ma R, Churilov L, Debono D, Nikfarjam M, Christophi C, Weinberg L (2019). The financial impact of postoperative complications following liver resection. Med (Baltim).

[34] SORAFENIB price [https://www.pbs.gov.au/medicine/item/9380q]

[35] LENVATINIB price [https://www.pbs.gov.au/medicine/item/10952K–11638 M]

[36] Reeve R, Srasuebkul P, Langton JM, Haas M, Viney R, Pearson S-A (2018). Health care use and costs at the end of life: a comparison of elderly australian decedents with and without a cancer history. BMC Palliat Care.

[37] McCaffrey N, Kaambwa B, Currow DC, Ratcliffe J (2016). Health-related quality of life measured using the EQ-5D-5L: South australian population norms. Health Qual Life Outcomes.

[38] Xiao Y, Howell J, van Gemert C, Thompson AJ, Seaman CP, McCulloch K, Scott N, Hellard ME (2020). Enhancing the hepatitis B care cascade in Australia: a cost-effectiveness model. J Viral Hepat.

[39] McPhail SM, Amarasena S, Stuart KA, Hayward K, Gupta R, Brain D, Hartel G, Rahman T, Clark PJ, Bernardes CM (2021). Assessment of health-related quality of life and health utilities in australian patients with cirrhosis. JGH Open.

[40] Kim HL, An J, Park JA, Park SH, Lim YS, Lee EK (2019). Magnetic resonance imaging is cost-effective for Hepatocellular Carcinoma Surveillance in high-risk patients with cirrhosis. Hepatology.

[41] Levy AR, Kowdley KV, Iloeje U, Tafesse E, Mukherjee J, Gish R, Bzowej N, Briggs AH (2008). The impact of chronic hepatitis B on quality of life: a multinational study of utilities from infected and uninfected persons. Value Health.

[42] Institute for Health Metrics and Evaluation (IHME). Global burden of Disease Study 2019 (GBD 2019) disability weights. In. Seattle. United States of America; 2020.

[43] The Pharmaceutical Benefits Advisory Committee Guidelines. :Overview and rationale of the economic evaluation. In.

[44] Wang S, Gum D, Merlin T (2018). Comparing the ICERs in Medicine reimbursement submissions to NICE and PBAC–Does the Presence of an explicit threshold affect the ICER proposed?. Value Health.

[45] Australian Institute of Health and Welfare. : BreastScreen Australia monitoring report 2021. In. Canberra; 2021.

[46] Goossens N, Singal AG, King LY, Andersson KL, Fuchs BC, Besa C, Taouli B, Chung RT, Hoshida Y (2017). Cost–effectiveness of risk score–stratified Hepatocellular Carcinoma Screening in patients with cirrhosis. Clin Transl Gastroenterol.

[47] Uyei J, Taddei TH, Kaplan DE, Chapko M, Stevens ER, Braithwaite RS (2019). Setting ambitious targets for surveillance and treatment rates among patients with hepatitis C related cirrhosis impacts the cost–effectiveness of hepatocellular cancer surveillance and substantially increases life expectancy: a modeling study. PLoS ONE.

[48] Briggs AH, Weinstein MC, Fenwick EA, Karnon J, Sculpher MJ, Paltiel AD, Force ISMGRPT (2012). Model parameter estimation and uncertainty: a report of the ISPOR–SMDM modeling Good Research Practices Task Force––6. Value Health.

[49] Carter HE, Jeffrey GP, Ramm GA, Gordon LG (2021). Cost–effectiveness of a serum biomarker test for risk–stratified liver Ultrasound Screening for Hepatocellular Carcinoma. Value Health.

[50] Robotin MC, Kansil M, Howard K, George J, Tipper S, Dore GJ, Levy M, Penman AG (2009). Antiviral therapy for hepatitis B–related liver cancer prevention is more cost–effective than cancer screening. J Hepatol.

