# Supplementary material for: Hepatocellular carcinoma surveillance based on the Australian Consensus Guidelines: a health economic modelling study
Source: BMC Health Serv Res. 2023 Apr 19;23:378. doi: 10.1186/s12913-023-09360-4 (PMC10116722; doi:10.1186/s12913-023-09360-4)
Supplement: Supplementary file 1 — Appendix A. Input paratemeters of the model. Table A1. Transition probabilities at 6-month interval, proportion values and discount rate used in the model. Table A2. Costs values (inflated to 2019-20 price)* at 6-month interval and Health state utility values (HSUV) used in the model. Table A3. Health price index, 2013/14 to 2019/20 (reference year 2019/20: 100) [30]. Table A5. Treatments for HCC at different BCLC stages (Triangular distribution for PSA) [32]. Table A6. Cumulative mortality rate of Cholangiocarcinoma at time of diagnosis [33]. Table A7. Cumulative mortality rate after liver transplant [12]. Table A8. Annual recurrence rate after liver resection [34]. Table A9. Cumulative recurrence rate after liver ablation [35]. Table A10. Treatments after HCC recurrence (Beta distribution for PSA with Standard deviation = 20% of the mean.Appendix B. Distribution of age at baseline in Australia, 2020. Table B1. Number and percentage of Australians by age. Figure B1. Distribution of age at baseline. Appendix C. Cost-effectiveness results of exclusive surveillance for non-cirrhotic CHB, compensated cirrhosis and decompensated cirrhosis population. Table C1. Cost-effectiveness of surveillance strategies on non-cirrhotic CHB people only. Table C2. Cost-effectiveness of surveillance strategies on compensated cirrhosis patients only.Table C3. Cost-effectiveness of surveillance strategies on decompensated cirrhosis patients only. Appendix D. Results of sensitivity and threshold analyses. Figure D2. Disease progression rates from CHB to compensated cirrhosis at different age ranges: (A) 20 to 80; (B) 30 to 80; (C) 40 to 80; (D) 50 to 80. Figure D3. Incremental cost-effectiveness scatterplot for Ultrasound+AFP versus status quo at real-world adherence rates, baseline population aged 40 to 80 years (green points represent optimal strategies, eclipse area represents 95% confidence interval). Figure D4. Incremental cost-effectiveness scatterplot for Ultrasound versus status qu [file 12913_2023_9360_MOESM1_ESM.docx]

Appendix A. Input paratemeters of the model

Table A1. Transition probabilities at 6-month interval, proportion values and discount rate used in the model

|  | Base-case value | Range for Sensitivity Analysis | Distribution for PSA | Source | |
| --- | --- | --- | --- | --- | --- |
| Disease progression |  |  |  |  | |
| CHB undertaking treatment to compensated cirrhosis | 0.0006 | 0.0003 – 0.0015 | Beta | [1] | |
| CHB not undertaking treatment to compensated cirrhosis | 0.0075 | 0.0002 – 0.0240 | Beta |  |  |
| CHB undertaking treatment to liver mass | 0.0003 | 0.0002 – 0.0008 | Beta |  |  |
| CHB not undertaking treatment to liver mass | 0.0013 | 0.0009 – 0.0038 | Beta |  |  |
| Compensated cirrhosis to decompensated cirrhosis | 0.0304 | 0.0150 – 0.0460 | Beta | [2] | |
| Compensated cirrhosis to liver mass | 0.0156 | 0.0080 – 0.0230 | Beta | [3] | |
| Decompensated cirrhosis to liver mass | 0.0379 | 0.0302 – 0.0454 | Beta | [4] | |
| Trans-arterial chemoembolisation to best supportive care for HCC | 0.3680 | 0.2940 – 0.4420 | Beta | Assumptions | |
| Selective internal radiation therapy to best supportive care for HCC |  |  |  |  |  |
| Systemic therapy to best supportive care for HCC | 0.4520 | 0.3620 – 0.5420 | Beta | [5] | |
| Probability of asymptomatic liver mass became symptomatic | 0.3680 | 0.2760 – 0.4590 | Beta | [6] | |
| Probability of liver mass larger than 10 mm | 0.930 | 0.800 – 0.980 | Beta | [7] | |
| Proportion of liver mass in CHB patients |  |  |  |  | |
| Benign | 0.120 | 0.070 – 0.170 | Beta | [8] | |
| HCC | 0.760 | 0.710 – 0.810 | Beta |  |  |
| Other types of liver cancer | 0.120 | complement of sum of all other liver mass probabilities | Beta |  |  |
| Proportion of liver mass in cirrhotic patients |  |  |  |  | |
| Benign | 0.167 | 0.117 – 0.217 | Beta | [9] | |
| HCC | 0.700 | 0.650 – 0.750 | Beta |  |  |
| Other types of liver cancer | 0.133 | complement of sum of all other liver mass probabilities | Beta |  |  |
| Probabilities of dying from |  |  |  |  | |
| Compensated cirrhosis | 0.020 | 0.005 – 0.050 | Beta | [10] | |
| Decompensated cirrhosis | 0.095 | 0.045 – 0.150 | Beta | [2] | |
| Best supportive care for HCC | 0.338 | 0.270 – 0.406 | Beta | [11] | |
| Liver transplant perioperative death | 0.030 | 0.020 – 0.040 | Beta | [12] | |
| Liver resection perioperative death | 0.048 | 0.038 – 0.058 | Beta | [13] | |
| Proportion of HCC by BCLC stages for Status Quo strategy |  |  |  |  | |
| Stage 0/A | 0.096 | 0.010– 0.300 | Beta | [14] | |
| Stage B | 0.237 | complement of sum of all other BCLC stages probabilities | Beta |  |  |
| Stage C | 0.500 | 0.300 – 0.650 | Beta |  |  |
| Stage D | 0.167 | 0.010 – 0.300 | Beta |  |  |
| Proportion of HCC by BCLC stages for 6-monthly ultrasound strategy |  |  |  |  | |
| Stage 0/A | 0.491 | 0.344 – 0.638 | Beta | [14] | |
| Stage B | 0.191 | complement of sum of all other BCLC stages probabilities | Beta |  |  |
| Stage C | 0.182 | 0.090 – 0.360 | Beta |  |  |
| Stage D | 0.136 | 0.030 – 0.300 | Beta |  |  |
| Proportion of HCC by BCLC stages for 6-monthly ultrasound+AFP strategy |  |  |  |  | |
| Stage 0/A | 0.618 | 0.402 – 0.803 | Beta | [15] | |
| Stage B | 0.191 | complement of sum of all other BCLC stages probabilities | Beta |  |  |
| Stage C | 0.129 | 0.050 – 0.290 | Beta |  |  |
| Stage D | 0.062 | 0.012 – 0.250 | Beta |  |  |
| Other input parameters |  |  |  |  | |
| Proportion of population of interest |  |  |  |  | |
| CHB | 0.150 | complement of sum of all other population of interest | NA | Assumption | |
| Compensated Cirrhosis | 0.750 | 0.600 – 0.800 | NA | Assumption | |
| Decompensated Cirrhosis | 0.100 | 0.000 – 0.200 | NA | Assumption | |
| Adherence rates |  |  |  |  | |
| After 1 year | 0.672 | NA | NA | [16] | |
| After 2 years | 0.470 | NA | NA |  |  |
| After 5 years | 0.400 | NA | NA |  |  |
| After 10 years | 0.239 | NA | NA |  |  |
| After 30 years | 0.000 | NA | NA | Assumption | |
| Discount rate | 0.050 | 0.030 – 0.070 | Uniform | [17] | |
| Proportion of CT scan being used for tumour diagnosis | 0.900 | 0.80 – 0.950 | NA | Assumption | |
| Proportion of MRI scan being used for tumour diagnosis | 0.100 | complement of “Proportion of CT scan being used for tumour diagnosis” | NA | Assumption | |
| Probability of indeterminate liver masses diagnosed by liver biopsy | 0.100 | 0.050 – 0.150 | NA | Assumption | |
| Proportion of CHB patients taking treatment | 0.093 | 0.050 – 0.150 | NA | [18] | |
| Proportion of CHB patients not taking treatment | 0.907 | complement of “Proportion of CHB patients taking treatment” | NA | [18] | |
| Specificity of surveillance using ultrasound | 0.910 | 0.865 – 0.955 | Beta | [19] | |
| Specificity of surveillance using ultrasound+AFP | 0.840 | 0.756 – 0.924 | Beta | [19] | |
| *AFP, alpha-fetoprotein; BCLC, Barcelona clinic liver cancer; CHB, chronic hepatitis B; CT, computed tomography; HCC, hepatocellular carcinoma; MRI, magnetic resonance imaging; PSA, probabilistic sensitivity analysis* | | | | |  |

Table A2. Costs values (inflated to 2019-20 price)^*^ at 6-month interval and Health state utility values (HSUV) used in the model

|  | Base-case value | Range for SA | Distribution for PSA | Source |
| --- | --- | --- | --- | --- |
| Cost (6-month cycle) |  |  |  |  |
| Base health states^†^ |  |  |  |  |
| CHB without treatment | 283.6 | 226.9 – 340.3 | Gamma | [1] |
| CHB with treatment | 2,029.0 | 1,623.2 – 2,434.8 | Gamma |  |
| Compensated cirrhosis | 2,120.7 | 1,696.6 – 2,544.8 | Gamma |  |
| Decompensated cirrhosis | 10,214.4 | 8,171.5 – 12,257.28 | Gamma |  |
| Screening and diagnosis |  |  | Gamma |  |
| Ultrasound | 111.3 | 89.0 – 139.0 | Gamma | MBS item 55036 [20] |
| AFP | 24.5 | 12.0 – 50.0 | Gamma | MBS item 66650 [20] |
| CT | 480.1 | 384.1 – 576.1 | Gamma | MBS item 56507 [20] |
| MRI | 550.0 | 440.0 – 660.0 | Gamma | MBS item 63545/63546 [20] |
| Biopsy | 257.7 | 206.2 – 309.2 | Gamma | MBS item 30409 & 20702 [20] |
| HCC treatments |  |  | Gamma |  |
| Resection^††^ | 19,645.1 | 15,716.1 – 23,574.1 | Gamma | [21] |
| Transplant^††^ | 192,880.0 | 173,592 – 231,456 | Gamma | [22] |
| Ablative therapies | 1,116.8 | 893.4 – 1,340.2 | Gamma | MBS item (30419/50950/ 50952) & 20799 & 51303 [20] |
| SIRT | 1,112.2 | 889.8 – 1334.6 | Gamma | MBS item 35406 & 20799 & 51303 [20] |
| TACE | 681.1 | 544.9 – 817.3 | Gamma | MBS item 34527 & 20799 [20] |
| Systemic therapy (sorafenib + levatinib)^†††^ | 36,108.7 | 28,887.0 – 43,330.4 | Gamma | [23, 24] |
| Best supportive care^†^ | 33,641.9 | 26,913.5 – 40,370.3 | Gamma | [25] |
| HSUVs |  |  |  |  |
| Compensated cirrhosis | 0.680 | 0.544 – 0.816 | Beta | [26] |
| Decompensated cirrhosis | 0.390 | 0.292 – 0.488 | Beta | [27] |
| After treatments for HCC |  |  | Beta |  |
| Liver transplant (in the first year) | 0.600 | 0.480 – 0.720 | Beta | [6] |
| Liver transplant (after the first year) | 0.850 | 0.750 – 0.950 | Beta | [6] |
| Liver resection | 0.700 | 0.525 – 0.875 | Beta | [28] |
| Liver ablation | 0.870 | 0.783 – 0.975 | Beta | [6] |
| SIRT | 0.500 | 0.300 – 0.700 | Beta | Assumption |
| TACE |  |  |  |  |
| Systemic therapy (sorafenib + levatinib) | 0.460 | 0.368 – 0.522 | Beta | [29] |
| Best supportive care | 0.200 | 0.050 – 0.350 | Beta | [28] |
| **Costs were reported in 2019 AUD and inflated using the total health price index and the Government final consumption expenditure on hospitals and nursing homes (Table A3) [30]*  *† Inflated using total health price index*  *†† Inflated using Government final consumption expenditure on hospitals and nursing homes*  *††† The number of Sorafenib/Levatinib tablets taken over 6-month cycle was multiplied with per-quantity-unit price, which was calculated from the Dispense Price for Maximum Quantity subtracted by the patient contributions and divided by the Maximum Quantity Units. Sorafenib and levatinib were assumed to be used 90% and 10% of the time, respectively.*  *AFP, alpha-fetoprotein; CHB, chronic hepatitis B; CT, computed tomography; HCC, hepatocellular carcinoma; MRI, magnetic resonance imaging; PSA, probabilistic sensitivity analysis; SIRT, selective internal radiation therapy; TACE, trans-arterial chemoembolisation*  Table A3. Health price index, 2013/14 to 2019/20 (reference year 2019/20: 100) [30]   \| Index \| 2013/14 \| 2014/15 \| 2015/16 \| 2016/17 \| 2017/18 \| 2018/19 \| 2019/20 \| \| --- \| --- \| --- \| --- \| --- \| --- \| --- \| --- \| \| Total health price index \| 89.3 \| 90.7 \| 92.6 \| 94.7 \| 96.2 \| 98.2 \| 100.0 \| \| Government final consumption expenditure on hospitals and nursing homes \| 86.9 \| 89.0 \| 90.6 \| 92.3 \| 94.7 \| 97.5 \| 100.0 \|   Table A4. Health state utility values (HSUV) for CHB people   \| Age (years) \| Population norm for EQ-5D [31] \| Disability weight for CHB in treatment [1] \| Disability weight for CHB not in treatment [1] \| HSUV for CHB in treatment \| HSUV for CHB not in treatment \| \| --- \| --- \| --- \| --- \| --- \| --- \| \| 40 - 44 \| 0.920 \| 0.012  (0.000 – 0.050) \| 0.051  (0.000 – 0.010) \| 0.908 \| 0.869 \| \| 45 - 64 \| 0.890 \| 0.878 \| 0.839 \| \| 65 - 74 \| 0.870 \| 0.858 \| 0.819 \| \| 75 + \| 0.830 \| 0.818 \| 0.779 \|   Table A5. Treatments for HCC at different BCLC stages (Triangular distribution for PSA) [32]   \|  \| BCLC stage 0/A (range for Sensitivity Analysis) \| BCLC stage B (range for Sensitivity Analysis) \| BCLC stage C (range for Sensitivity Analysis) \| BCLC stage D (range for Sensitivity Analysis) \| \| --- \| --- \| --- \| --- \| --- \| \| Liver transplant \| 0.029 (0.010 – 0.090) \| 0.033 (0.010 – 0.090) \| 0.020 (0.010 – 0.090) \| 0.164 (0.100 – 0.200) \| \| Resection \| 0.422 (complement of sum of all other treatments probabilities) \| 0.339 (complement of sum of all other treatments probabilities) \| 0.170 (complement of sum of all other treatments probabilities) \| 0.017 (0.000 – 0.040) \| \| Ablative therapies \| 0.347 (0.295 – 0.399) \| 0.083 (0.033 – 0.183) \| 0.042 (0.022 – 0.062) \| 0.026 (0.010 – 0.050) \| \| TACE \| 0.193 (0.154 – 0.232) \| 0.496 (0.450 – 0.550) \| 0.193 (0.153 – 0.213) \| 0.043 (0.023 – 0.063) \| \| SIRT \| 0.000 (0.000 – 0.040) \| 0.029 (0.010 – 0.050) \| 0.168 (0.128 – 0.188) \| 0.026 (0.010 – 0.050) \| \| Targeted therapy \| 0.006 (0.000 – 0.040) \| 0.008 (0.000 – 0.040) \| 0.260 (0.225 – 0.275) \| 0.009 (0.000 – 0.040) \| \| Best supportive care \| 0.004 (0.000 – 0.040) \| 0.012 (0.000 – 0.040) \| 0.148 (0.098 – 0.168) \| 0.716 (complement of sum of all other treatments probabilities) \| \| *BCLC, Barcelona clinic liver cancer; SIRT, selective internal radiation therapy; TACE, trans-arterial chemoembolisation* \| \| \| \| \|   Table A6. Cumulative mortality rate of Cholangiocarcinoma at time of diagnosis [33]   \| Years after diagnosis \| Cumulative mortality rate \| \| --- \| --- \| \| 1 \| 0.423 \| \| 2 \| 0.628 \| \| 3 \| 0.728 \| \| 4 \| 0.799 \| \| 5 \| 0.823 \| \| 6 \| 0.91 \| \| 10 \| 1 \|   Table A7. Cumulative mortality rate after liver transplant [12]   \| Time post transplant (years) \| Survival rate \| Cumulative mortality rate \| \| --- \| --- \| --- \| \| 0 \| 1.00 \| 0.00 \| \| 1 \| 0.94 \| 0.06 \| \| 5 \| 0.78 \| 0.22 \| \| 10 \| 0.68 \| 0.32 \| \| 15 \| 0.66 \| 0.34 \| \| 20 \| 0.61 \| 0.39 \| \| 25 \| 0.54 \| 0.46 \| \| 30 \| 0.00 \| 1.00 \|   Table A8. Annual recurrence rate after liver resection [34]   \| Time post resection (years) \| Recurrence rate (stage 0/A) \| Recurrence rate (stage B+C+D) \| \| --- \| --- \| --- \| \| 0 \| 0.0000 \| 0.0000 \| \| 1 \| 0.2130 \| 0.3830 \| \| 2 \| 0.1310 \| 0.1360 \| \| 3 \| 0.0460 \| 0.0450 \| \| 4 \| 0.0360 \| 0.0190 \| \| 5 \| 0.0180 \| 0.0001 \| \| 6 (onwards) \| 0.0015 \| 0.0068 \|   Table A9. Cumulative recurrence rate after liver ablation [35]   \| Time post ablation (years) \| Cumulative recurrence rate \| \| --- \| --- \| \| 0 \| 0.000 \| \| 1 \| 0.374 \| \| 2 \| 0.541 \| \| 3 \| 0.710 \| \| 5 \| 0.795 \| \| 15 \| 1.000 \|   Table A10. Treatments after HCC recurrence (Beta distribution for PSA with Standard deviation = 20% of the mean)   \| Treatment  Recur after \| Resection \| Transplant \| RFA \| TACE \| SIRT \| Sorafenib \| Best supportive care \| \| --- \| --- \| --- \| --- \| --- \| --- \| --- \| --- \| \| Resection [36] \| 0.1311 \| 0.1038 \| 0.2404 \| 0.3279 \| 0.0000 \| 0.0383 \| 0.1585 \| \| Liver transplant [37] \| 0.0900 \| 0.0000 \| 0.0000 \| 0.2500 \| 0.0000 \| 0.4800 \| 0.1800 \| \| Liver ablation [38] \| 0.0300 \| 0.1010 \| 0.7090 \| 0.0450 \| 0.0550 \| 0.0100 \| 0.0500 \| \| *Sensitivity analyses were conducted on these transition parameters and very little difference was made to the outcomes.* \| \| \| \| \| \| \| \| | | | | |

Appendix B. Distribution of age at baseline in Australia, 2020


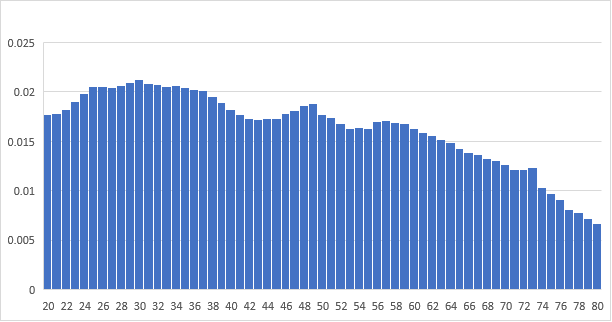
Table B1. Number and percentage of Australians by age

| Age | No. of people | Age distribution |
| --- | --- | --- |
| 20 | 326,630 | 0.018 |
| 21 | 329,353 | 0.018 |
| 22 | 337,043 | 0.018 |
| 23 | 352,106 | 0.019 |
| 24 | 366,225 | 0.020 |
| 25 | 379,053 | 0.020 |
| 26 | 379,239 | 0.021 |
| 27 | 378,582 | 0.020 |
| 28 | 381,346 | 0.021 |
| 29 | 388,087 | 0.021 |
| 30 | 392,965 | 0.021 |
| 31 | 385,187 | 0.021 |
| 32 | 383,618 | 0.021 |
| 33 | 379,329 | 0.021 |
| 34 | 382,306 | 0.021 |
| 35 | 377,316 | 0.020 |
| 36 | 374,850 | 0.020 |
| 37 | 371,643 | 0.020 |
| 38 | 360,277 | 0.019 |
| 39 | 350,448 | 0.019 |
| 40 | 336,461 | 0.018 |
| 41 | 326,749 | 0.018 |
| 42 | 320,186 | 0.017 |
| 43 | 317,293 | 0.017 |
| 44 | 319,182 | 0.017 |
| 45 | 320,111 | 0.017 |
| 46 | 328,662 | 0.018 |
| 47 | 334,177 | 0.018 |
| 48 | 345,145 | 0.019 |
| 49 | 348,546 | 0.019 |
| 50 | 327,042 | 0.018 |
| 51 | 321,316 | 0.017 |
| 52 | 310,866 | 0.017 |
| 53 | 302,272 | 0.016 |
| 54 | 302,997 | 0.016 |
| 55 | 302,219 | 0.016 |
| 56 | 313,800 | 0.017 |
| 57 | 316,231 | 0.017 |
| 58 | 313,166 | 0.017 |
| 59 | 310,983 | 0.017 |
| 60 | 301,089 | 0.016 |
| 61 | 293,072 | 0.016 |
| 62 | 288,023 | 0.016 |
| 63 | 279,935 | 0.015 |
| 64 | 275,316 | 0.015 |
| 65 | 264,195 | 0.014 |
| 66 | 256,694 | 0.014 |
| 67 | 252,727 | 0.014 |
| 68 | 244,455 | 0.013 |
| 69 | 241,205 | 0.013 |
| 70 | 234,518 | 0.013 |
| 71 | 225,551 | 0.012 |
| 72 | 225,264 | 0.012 |
| 73 | 228,682 | 0.012 |
| 74 | 191,094 | 0.010 |
| 75 | 178,984 | 0.010 |
| 76 | 168,257 | 0.009 |
| 77 | 149,228 | 0.008 |
| 78 | 145,242 | 0.008 |
| 79 | 132,330 | 0.007 |
| 80 | 123,759 | 0.007 |

Figure B1. Distribution of age at baseline

The age distribution of our cohort was assumed to be similar to the overall age distribution of Australia. Total number of people at each age was obtained from the National Population by age, Australian Bureau of Statistics (ABS) [39] and the proportion of population for each age was calculated to derive the age distribution of the population. The oldest starting age for surveillance was chosen as 80 years as curative treatments such as liver resection [40, 41] and ablative therapies [42, 43] were shown to be safe and feasible for octogenarians.

Appendix C. Cost-effectiveness results of exclusive surveillance for non-cirrhotic CHB, compensated cirrhosis and decompensated cirrhosis population

Table C1. Cost-effectiveness of surveillance strategies on non-cirrhotic CHB people only

| Cohort starting age | Surveillance strategy | Cost | Incr. cost vs next most cost-effective alternative | QALY | Incr. QALY vs next most cost-effective alternative | ICER vs next most cost-effective alternative | ICER vs status quo | Domiance |  |
| --- | --- | --- | --- | --- | --- | --- | --- | --- | --- |
| 20-80 | Status quo (1) | 23,192 |  | 11.96 |  |  |  |  |  |
|  | Ultrasound_real-world adherence (2) | 24,479 | 1,288 | 11.97 | 0.01 | 112,734 | 112,734 |  |  |
|  | Ultrasound+AFP_real-world adherence (3) | 24,922 | 442 | 11.97 | 0.00 | 179,252 | 124,546 | Extendedly dominated^†^ |  |
|  | Ultrasound_full adherence (4) | 27,880 | 2,959 | 12.00 | 0.02 | 123,002 | 123,567 | Extendedly dominated |  |
|  | Ultrasound+AFP_full adherence (5) | 29,506 | 5,027 | 12.01 | 0.04 | 113,285 | 113,172 |  |  |
| 30-80 | Status quo (1) | 21,906 |  | 11.41 |  |  |  |  |  |
|  | Ultrasound_real-world adherence (2) | 23,208 | 1,302 | 11.42 | 0.01 | 102,469 | 102,469 |  |  |
|  | Ultrasound+AFP_real-world adherence (3) | 23,652 | 445 | 11.42 | 0.00 | 222,184 | 118,760 | Extendedly dominated |  |
|  | Ultrasound_full adherence (4) | 26,432 | 2,780 | 11.45 | 0.02 | 122,766 | 121,189 | Extendedly dominated |  |
|  | Ultrasound+AFP_full adherence (5) | 27,989 | 4,782 | 11.46 | 0.04 | 116,727 | 113,353 |  |  |
| 40-80 | Status quo (1) | 20,331 |  | 10.69 |  |  |  |  |  |
|  | Ultrasound_real-world adherence (2) | 21,559 | 1,228 | 10.70 | 0.01 | 128,351 | 128,351 | Extendedly dominated |  |
|  | Ultrasound+AFP_real-world adherence (3) | 21,998 | 439 | 10.70 | 0.00 | 217,238 | 143,855 | Extendedly dominated |  |
|  | Ultrasound_full adherence (4) | 24,551 | 2,553 | 10.72 | 0.02 | 134,020 | 137,740 | Extendedly dominated |  |
|  | Ultrasound+AFP_full adherence (5) | 26,035 | 5,704 | 10.74 | 0.05 | 125,286 | 125,286 |  |  |
| 50-80 | Status quo (1) | 18,538 |  | 9.85 |  |  |  |  |  |
|  | Ultrasound_real-world adherence (2) | 19,776 | 1,238 | 9.86 | 0.01 | 155,578 | 155,578 |  |  |
|  | Ultrasound+AFP_real-world adherence (3) | 20,212 | 437 | 9.86 | 0.00 | 191,668 | 163,612 | Extendedly dominated |  |
|  | Ultrasound_full adherence (4) | 22,375 | 2,163 | 9.87 | 0.01 | 319,111 | 225,566 | Extendedly dominated |  |
|  | Ultrasound+AFP_full adherence (5) | 23,752 | 3,976 | 9.88 | 0.02 | 186,229 | 177,909 |  |  |
| 20-70 | Status quo (1) | 24,121 |  | 12.42 |  |  |  |  |  |
|  | Ultrasound_real-world adherence (2) | 25,432 | 1,311 | 12.43 | 0.01 | 110,165 | 110,165 | Extendedly dominated |  |
|  | Ultrasound+AFP_real-world adherence (3) | 25,900 | 468 | 12.43 | 0.00 | 133,571 | 115,492 | Extendedly dominated |  |
|  | Ultrasound_full adherence (4) | 29,022 | 3,122 | 12.46 | 0.03 | 118,320 | 117,278 | Extendedly dominated |  |
|  | Ultrasound+AFP_full adherence (5) | 30,710 | 6,588 | 12.48 | 0.06 | 107,902 | 107,902 |  |  |
| 30-70 | Status quo (1) | 23,189 |  | 11.99 |  |  |  |  |  |
|  | Ultrasound_real-world adherence (2) | 24,482 | 1,292 | 12.01 | 0.01 | 122,364 | 122,364 | Extendedly dominated |  |
|  | Ultrasound+AFP_real-world adherence (3) | 24,951 | 469 | 12.01 | 0.00 | 126,982 | 123,561 | Extendedly dominated |  |
|  | Ultrasound_full adherence (4) | 27,978 | 3,027 | 12.03 | 0.02 | 126,781 | 125,577 | Extendedly dominated |  |
|  | Ultrasound+AFP_full adherence (5) | 29,656 | 6,467 | 12.05 | 0.06 | 110,698 | 110,698 |  |  |
| 40-70 | Status quo (1) | 21,803 |  | 11.35 |  |  |  |  |  |
|  | Ultrasound_real-world adherence (2) | 23,102 | 1,299 | 11.36 | 0.01 | 113,669 | 113,669 |  |  |
|  | Ultrasound+AFP_real-world adherence (3) | 23,564 | 461 | 11.37 | 0.00 | 124,639 | 116,353 | Extendedly dominated |  |
|  | Ultrasound_full adherence (4) | 26,324 | 2,761 | 11.39 | 0.02 | 136,071 | 127,649 | Extendedly dominated |  |
|  | Ultrasound+AFP_full adherence (5) | 27,895 | 4,793 | 11.41 | 0.04 | 116,981 | 116,259 |  |  |
| 50-70 | Status quo (1) | 20,308 |  | 10.65 |  |  |  |  |  |
|  | Ultrasound_real-world adherence (2) | 21,580 | 1,272 | 10.66 | 0.01 | 136,503 | 136,503 | Extendedly dominated |  |
|  | Ultrasound+AFP_real-world adherence (3) | 22,040 | 460 | 10.66 | 0.00 | 135,917 | 136,347 | Extendedly dominated |  |
|  | Ultrasound_full adherence (4) | 24,511 | 2,471 | 10.67 | 0.02 | 153,372 | 145,867 | Extendedly dominated |  |
|  | Ultrasound+AFP_full adherence (5) | 25,994 | 5,685 | 10.69 | 0.04 | 128,659 | 128,659 |  |  |
| 20-60 | Status quo (1) | 25,355 |  | 12.98 |  |  |  |  |  |
|  | Ultrasound_real-world adherence (2) | 26,667 | 1,312 | 12.99 | 0.01 | 111,716 | 111,716 | Extendedly dominated |  |
|  | Ultrasound+AFP_real-world adherence (3) | 27,139 | 472 | 12.99 | 0.00 | 120,119 | 113,823 | Extendedly dominated |  |
|  | Ultrasound_full adherence (4) | 30,429 | 3,290 | 13.02 | 0.03 | 113,948 | 113,904 | Extendedly dominated |  |
|  | Ultrasound+AFP_full adherence (5) | 32,208 | 6,854 | 13.05 | 0.07 | 102,577 | 102,577 |  |  |
| 30-60 | Status quo (1) | 24,602 |  | 12.61 |  |  |  |  |  |
|  | Ultrasound_real-world adherence (2) | 25,915 | 1,313 | 12.62 | 0.01 | 121,012 | 121,012 | Extendedly dominated |  |
|  | Ultrasound+AFP_real-world adherence (3) | 26,385 | 471 | 12.62 | 0.00 | 120,871 | 120,975 | Extendedly dominated |  |
|  | Ultrasound_full adherence (4) | 29,540 | 3,155 | 12.65 | 0.02 | 135,114 | 129,642 | Extendedly dominated |  |
|  | Ultrasound+AFP_full adherence (5) | 31,287 | 6,686 | 12.67 | 0.06 | 115,045 | 115,045 |  |  |
| 40-60 | Status quo (1) | 23,369 |  | 12.03 |  |  |  |  |  |
|  | Ultrasound_real-world adherence (2) | 24,690 | 1,322 | 12.04 | 0.01 | 112,085 | 112,085 | Extendedly dominated |  |
|  | Ultrasound+AFP_real-world adherence (3) | 25,157 | 467 | 12.05 | 0.00 | 134,893 | 117,263 | Extendedly dominated |  |
|  | Ultrasound_full adherence (4) | 28,139 | 2,981 | 12.07 | 0.03 | 110,671 | 113,054 | Extendedly dominated |  |
|  | Ultrasound+AFP_full adherence (5) | 29,823 | 6,455 | 12.09 | 0.06 | 105,738 | 105,738 |  |  |
| 50-60 | Status quo (1) | 22,013 |  | 11.44 |  |  |  |  |  |
|  | Ultrasound_real-world adherence (2) | 23,316 | 1,303 | 11.45 | 0.01 | 109,727 | 109,727 |  | |
|  | Ultrasound+AFP_real-world adherence (3) | 23,783 | 467 | 11.46 | 0.00 | 137,484 | 115,895 | Extendedly dominated | |
|  | Ultrasound_full adherence (4) | 26,580 | 2,797 | 11.48 | 0.02 | 141,531 | 130,357 | Extendedly dominated | |
|  | Ultrasound+AFP_full adherence (5) | 28,167 | 4,850 | 11.49 | 0.04 | 123,095 | 119,999 |  |  |

*AFP; Alpha-Fetoprotein; HCC, hepatocellular carcinoma; ICER, Incremental cost-effectiveness ratio; Incr., incremental; QALY, quality adjusted life years*

† *A strategy with a higher ICER (relative to the next alternative) and lower QALY than the alternative was extendedly dominated by the alternative*

Table C2. Cost-effectiveness of surveillance strategies on compensated cirrhosis patients only

| Cohort starting age | Surveillance strategy | Cost | Incr. cost vs next most cost-effective alternative | QALY | Incr. QALY vs next most cost-effective alternative | ICER vs next most cost-effective alternative | ICER vs status quo | Domiance | |
| --- | --- | --- | --- | --- | --- | --- | --- | --- | --- |
| 20-80 | Status quo (1) | 60,033 |  | 4.71 |  |  |  |  | |
|  | Ultrasound_real-world adherence (2) | 61,236 | 1,203 | 4.76 | 0.04 | 27,053 | 27,053 | Extendedly dominated^†^ | |
|  | Ultrasound+AFP_real-world adherence (3) | 61,710 | 1,677 | 4.78 | 0.07 | 25,331 | 25,331 |  | |
|  | Ultrasound_full adherence (4) | 62,321 | 611 | 4.79 | 0.01 | 59,393 | 29,913 | Extendedly dominated | |
|  | Ultrasound+AFP_full adherence (5) | 63,163 | 1,454 | 4.84 | 0.06 | 25,543 | 25,429 |  | |
| 30-80 | Status quo (1) | 59,184 |  | 4.66 |  |  |  |  | |
|  | Ultrasound_real-world adherence (2) | 60,450 | 1,266 | 4.71 | 0.05 | 27,763 | 27,763 | Extendedly dominated | |
|  | Ultrasound+AFP_real-world adherence (3) | 60,923 | 473 | 4.73 | 0.02 | 22,673 | 26,165 | Extendedly dominated | |
|  | Ultrasound_full adherence (4) | 61,503 | 580 | 4.74 | 0.01 | 50,010 | 29,709 | Extendedly dominated | |
|  | Ultrasound+AFP_full adherence (5) | 62,326 | 3,142 | 4.79 | 0.12 | 25,719 | 25,719 |  | |
| 40-80 | Status quo (1) | 58,187 |  | 4.60 |  |  |  |  | |
|  | Ultrasound_real-world adherence (2) | 59,360 | 1,173 | 4.64 | 0.04 | 29,214 | 29,214 | Extendedly dominated | |
|  | Ultrasound+AFP_real-world adherence (3) | 59,794 | 433 | 4.66 | 0.02 | 21,353 | 26,575 | Extendedly dominated | |
|  | Ultrasound_full adherence (4) | 60,261 | 467 | 4.67 | 0.00 | 113,466 | 32,119 | Extendedly dominated | |
|  | Ultrasound+AFP_full adherence (5) | 60,957 | 2,769 | 4.71 | 0.10 | 26,565 | 26,565 |  | |
| 50-80 | Status quo (1) | 56,380 |  | 4.49 |  |  |  |  | |
|  | Ultrasound_real-world adherence (2) | 57,554 | 1,174 | 4.53 | 0.04 | 30,963 | 30,963 | Extendedly dominated | |
|  | Ultrasound+AFP_real-world adherence (3) | 57,972 | 418 | 4.55 | 0.02 | 23,715 | 28,664 | Extendedly dominated | |
|  | Ultrasound_full adherence (4) | 58,425 | 454 | 4.55 | 0.01 | 75,650 | 33,242 | Extendedly dominated | |
|  | Ultrasound+AFP_full adherence (5) | 59,026 | 2,646 | 4.59 | 0.10 | 27,517 | 27,517 |  | |
| 20-70 | Status quo (1) | 61,360 |  | 4.79 |  |  |  |  | |
|  | Ultrasound_real-world adherence (2) | 62,613 | 1,253 | 4.84 | 0.05 | 26,357 | 26,357 | Extendedly dominated | |
|  | Ultrasound+AFP_real-world adherence (3) | 63,099 | 487 | 4.86 | 0.02 | 21,415 | 24,759 | Extendedly dominated | |
|  | Ultrasound_full adherence (4) | 63,666 | 567 | 4.87 | 0.01 | 50,402 | 28,297 | Extendedly dominated | |
|  | Ultrasound+AFP_full adherence (5) | 64,490 | 3,130 | 4.92 | 0.13 | 24,153 | 24,153 |  | |
| 30-70 | Status quo (1) | 60,802 |  | 4.77 |  |  |  |  | |
|  | Ultrasound_real-world adherence (2) | 62,009 | 1,207 | 4.81 | 0.04 | 26,875 | 26,875 | Extendedly dominated | |
|  | Ultrasound+AFP_real-world adherence (3) | 62,492 | 1,690 | 4.83 | 0.07 | 25,301 | 25,301 |  | |
|  | Ultrasound_full adherence (4) | 63,095 | 603 | 4.84 | 0.01 | 96,247 | 31,381 | Extendedly dominated | |
|  | Ultrasound+AFP_full adherence (5) | 63,920 | 1,428 | 4.88 | 0.05 | 26,940 | 26,026 |  | |
| 40-70 | Status quo (1) | 60,105 |  | 4.71 |  |  |  |  | |
|  | Ultrasound_real-world adherence (2) | 61,288 | 1,183 | 4.75 | 0.04 | 29,727 | 29,727 | Extendedly dominated | |
|  | Ultrasound+AFP_real-world adherence (3) | 61,762 | 474 | 4.77 | 0.02 | 21,941 | 26,988 | Extendedly dominated | |
|  | Ultrasound_full adherence (4) | 62,241 | 479 | 4.78 | 0.01 | 51,863 | 30,240 | Extendedly dominated | |
|  | Ultrasound+AFP_full adherence (5) | 63,053 | 2,948 | 4.83 | 0.12 | 25,513 | 25,513 |  | |
| 50-70 | Status quo (1) | 58,968 |  | 4.64 |  |  |  |  | |
|  | Ultrasound_real-world adherence (2) | 60,097 | 1,128 | 4.68 | 0.04 | 30,522 | 30,522 | Extendedly dominated | |
|  | Ultrasound+AFP_real-world adherence (3) | 60,557 | 460 | 4.70 | 0.02 | 21,751 | 27,330 | Extendedly dominated | |
|  | Ultrasound_full adherence (4) | 61,085 | 529 | 4.70 | 0.01 | 82,895 | 32,824 | Extendedly dominated | |
|  | Ultrasound+AFP_full adherence (5) | 61,835 | 2,866 | 4.74 | 0.11 | 26,972 | 26,972 |  | |
| 20-60 | Status quo (1) | 62,346 |  | 4.85 |  |  |  |  | |
|  | Ultrasound_real-world adherence (2) | 63,564 | 1,218 | 4.90 | 0.05 | 27,040 | 27,040 | Extendedly dominated | |
|  | Ultrasound+AFP_real-world adherence (3) | 64,060 | 495 | 4.92 | 0.02 | 21,787 | 25,278 | Extendedly dominated | |
|  | Ultrasound_full adherence (4) | 64,600 | 540 | 4.93 | 0.01 | 45,445 | 28,286 | Extendedly dominated | |
|  | Ultrasound+AFP_full adherence (5) | 65,496 | 3,150 | 4.98 | 0.13 | 24,259 | 24,259 |  | |
| 30-60 | Status quo (1) | 61,915 |  | 4.83 |  |  |  |  | |
|  | Ultrasound_real-world adherence (2) | 63,129 | 1,215 | 4.87 | 0.04 | 27,364 | 27,364 | Extendedly dominated | |
|  | Ultrasound+AFP_real-world adherence (3) | 63,622 | 493 | 4.90 | 0.02 | 21,901 | 25,526 | Extendedly dominated | |
|  | Ultrasound_full adherence (4) | 64,212 | 590 | 4.91 | 0.01 | 44,704 | 28,687 | Extendedly dominated | |
|  | Ultrasound+AFP_full adherence (5) | 65,079 | 3,164 | 4.96 | 0.13 | 24,348 | 24,348 |  | |
| 40-60 | Status quo (1) | 61,453 |  | 4.79 |  |  |  |  | |
|  | Ultrasound_real-world adherence (2) | 62,659 | 1,206 | 4.84 | 0.04 | 27,433 | 27,433 | Extendedly dominated | |
|  | Ultrasound+AFP_real-world adherence (3) | 63,145 | 486 | 4.86 | 0.02 | 22,005 | 25,619 | Extendedly dominated | |
|  | Ultrasound_full adherence (4) | 63,703 | 558 | 4.87 | 0.01 | 47,907 | 28,960 | Extendedly dominated | |
|  | Ultrasound+AFP_full adherence (5) | 64,551 | 3,098 | 4.92 | 0.12 | 24,984 | 24,984 |  | |
| 50-60 | Status quo (1) | 60,431 |  | 4.73 |  |  |  |  | |
|  | Ultrasound_real-world adherence (2) | 61,634 | 1,203 | 4.78 | 0.04 | 27,438 | 27,438 | Extendedly dominated |  |
|  | Ultrasound+AFP_real-world adherence (3) | 62,120 | 486 | 4.80 | 0.02 | 22,246 | 25,711 | Extendedly dominated |  |
|  | Ultrasound_full adherence (4) | 62,666 | 546 | 4.81 | 0.01 | 52,999 | 29,410 | Extendedly dominated |  |
|  | Ultrasound+AFP_full adherence (5) | 63,475 | 3,044 | 4.85 | 0.12 | 25,308 | 25,308 |  | |

*AFP; Alpha-Fetoprotein; HCC, hepatocellular carcinoma; ICER, Incremental cost-effectiveness ratio; Incr., incremental; QALY, quality adjusted life years*

† *A strategy with a higher ICER (relative to the next alternative) and lower QALY than the alternative was extendedly dominated by the alternative*

Table C3. Cost-effectiveness of surveillance strategies on decompensated cirrhosis patients only

| Cohort starting age | Surveillance strategy | Cost | Incr. cost vs next most cost-effective alternative | QALY | Incr. QALY vs next most cost-effective alternative | ICER vs next most cost-effective alternative | ICER vs status quo | Domiance |
| --- | --- | --- | --- | --- | --- | --- | --- | --- |
| 20-80 | Status quo (1) | 84,434 |  | 1.58 |  |  |  |  |
|  | Ultrasound_real-world adherence (2) | 85,881 | 1,447 | 1.63 | 0.05 | 29,604 | 29,604 | Extendedly dominated^†^ |
|  | Ultrasound+AFP_real-world adherence (3) | 86,231 | 350 | 1.65 | 0.02 | 22,158 | 27,785 | Extendedly dominated |
|  | Ultrasound_full adherence (4) | 86,386 | 155 | 1.65 | 0.00 | 37,678 | 28,375 | Extendedly dominated |
|  | Ultrasound+AFP_full adherence (5) | 86,773 | 2,339 | 1.67 | 0.09 | 26,669 | 26,669 |  |
| 30-80 | Status quo (1) | 83,855 |  | 1.57 |  |  |  |  |
|  | Ultrasound_real-world adherence (2) | 85,243 | 1,389 | 1.62 | 0.05 | 28,864 | 28,864 | Extendedly dominated |
|  | Ultrasound+AFP_real-world adherence (3) | 85,708 | 465 | 1.64 | 0.02 | 25,530 | 27,948 | Extendedly dominated |
|  | Ultrasound_full adherence (4) | 85,723 | 15 | 1.64 | 0.00 | 16,440 | 27,797 | Extendedly dominated |
|  | Ultrasound+AFP_full adherence (5) | 86,224 | 2,369 | 1.66 | 0.09 | 26,860 | 26,860 |  |
| 40-80 | Status quo (1) | 83,383 |  | 1.56 |  |  |  |  |
|  | Ultrasound_real-world adherence (2) | 84,830 | 1,447 | 1.61 | 0.05 | 29,625 | 29,625 | Extendedly dominated |
|  | Ultrasound+AFP_real-world adherence (3) | 85,227 | 397 | 1.63 | 0.02 | 23,897 | 28,173 | Extendedly dominated |
|  | Ultrasound_full adherence (4) | 85,352 | 125 | 1.63 | 0.00 | 40,239 | 28,719 | Extendedly dominated |
|  | Ultrasound+AFP_full adherence (5) | 85,771 | 2,387 | 1.65 | 0.09 | 27,323 | 27,323 |  |
| 50-80 | Status quo (1) | 82,448 |  | 1.54 |  |  |  |  |
|  | Ultrasound_real-world adherence (2) | 83,836 | 1,388 | 1.59 | 0.05 | 29,741 | 29,741 | Extendedly dominated |
|  | Ultrasound+AFP_real-world adherence (3) | 84,271 | 435 | 1.61 | 0.02 | 25,305 | 28,547 | Extendedly dominated |
|  | Ultrasound_full adherence (4) | 84,308 | 37 | 1.61 | 0.00 | 59,300 | 28,841 | Extendedly dominated |
|  | Ultrasound+AFP_full adherence (5) | 84,765 | 2,317 | 1.63 | 0.08 | 27,672 | 27,672 |  |
| 20-70 | Status quo (1) | 85,070 |  | 1.59 |  |  |  |  |
|  | Ultrasound_real-world adherence (2) | 86,475 | 1,405 | 1.64 | 0.05 | 28,962 | 28,962 | Extendedly dominated |
|  | Ultrasound+AFP_real-world adherence (3) | 86,951 | 476 | 1.66 | 0.02 | 25,386 | 27,965 | Extendedly dominated |
|  | Ultrasound_full adherence (4) | 86,921 | -29 | 1.66 | -0.00 | 18,278 | 28,203 | Extendedly dominated |
|  | Ultrasound+AFP_full adherence (5) | 87,446 | 2,377 | 1.68 | 0.09 | 27,269 | 27,269 |  |
| 30-70 | Status quo (1) | 84,831 |  | 1.59 |  |  |  |  |
|  | Ultrasound_real-world adherence (2) | 86,252 | 1,421 | 1.64 | 0.05 | 29,610 | 29,610 | Extendedly dominated |
|  | Ultrasound+AFP_real-world adherence (3) | 86,702 | 450 | 1.66 | 0.02 | 24,837 | 28,301 | Extendedly dominated |
|  | Ultrasound_full adherence (4) | 86,602 | -100 | 1.65 | -0.00 | 39,186 | 27,865 | Extendedly dominated |
|  | Ultrasound+AFP_full adherence (5) | 87,105 | 2,274 | 1.67 | 0.09 | 26,664 | 26,664 |  |
| 40-70 | Status quo (1) | 84,401 |  | 1.58 |  |  |  |  |
|  | Ultrasound_real-world adherence (2) | 85,765 | 1,364 | 1.63 | 0.05 | 28,698.75 | 28,699 | Extendedly dominated |
|  | Ultrasound+AFP_real-world adherence (3) | 86,208 | 443 | 1.65 | 0.02 | 24,911.20 | 27,667 | Extendedly dominated |
|  | Ultrasound_full adherence (4) | 86,254 | 46 | 1.65 | 0.00 | 129,405.62 | 28,221 | Extendedly dominated |
|  | Ultrasound+AFP_full adherence (5) | 86,729 | 2,328 | 1.67 | 0.09 | 26,969.58 | 26,970 |  |
| 50-70 | Status quo (1) | 83,778 |  | 1.57 |  |  |  |  |
|  | Ultrasound_real-world adherence (2) | 85,121 | 1,343 | 1.62 | 0.05 | 28,972 | 28,972 | Extendedly dominated |
|  | Ultrasound+AFP_real-world adherence (3) | 85,589 | 468 | 1.63 | 0.02 | 25,715 | 28,054 | Extendedly dominated |
|  | Ultrasound_full adherence (4) | 85,493 | -96 | 1.63 | -0.00 | 49,449 | 27,388 | Extendedly dominated |
|  | Ultrasound+AFP_full adherence (5) | 85,984 | 2,206 | 1.65 | 0.08 | 26,554 | 26,554 |  |
| 20-60 | Status quo (1) | 85,355 |  | 1.60 |  |  |  |  |
|  | Ultrasound_real-world adherence (2) | 86,865 | 1,510 | 1.65 | 0.05 | 29,698 | 29,698 | Extendedly dominated |
|  | Ultrasound+AFP_real-world adherence (3) | 87,336 | 471 | 1.67 | 0.02 | 24,663 | 28,323 | Extendedly dominated |
|  | Ultrasound_full adherence (4) | 87,240 | -96 | 1.67 | -0.00 | 45,668 | 27,786 | Extendedly dominated |
|  | Ultrasound+AFP_full adherence (5) | 87,770 | 2,416 | 1.69 | 0.09 | 26,777 | 26,777 |  |
| 30-60 | Status quo (1) | 85,313 |  | 1.60 |  |  |  |  |
|  | Ultrasound_real-world adherence (2) | 86,798 | 1,485 | 1.65 | 0.05 | 29,592 | 29,592 | Extendedly dominated |
|  | Ultrasound_full adherence (4) | 87,216 | 418 | 1.67 | 0.02 | 23,241 | 27,916 | Extendedly dominated |
|  | Ultrasound+AFP_real-world adherence (3) | 87,235 | 18 | 1.67 | -0.00 | -55,668 | 28,324 | Absolutely dominated^§^ |
|  | Ultrasound+AFP_full adherence (5) | 87,717 | 2,404 | 1.69 | 0.09 | 26,942 | 26,942 |  |
| 40-60 | Status quo (1) | 85,099 |  | 1.59 |  |  |  |  |
|  | Ultrasound_real-world adherence (2) | 86,542 | 1,443 | 1.64 | 0.05 | 29,235 | 29,235 | Extendedly dominated |
|  | Ultrasound+AFP_real-world adherence (3) | 86,980 | 439 | 1.66 | 0.02 | 24,810 | 28,067 | Extendedly dominated |
|  | Ultrasound_full adherence (4) | 86,937 | -44 | 1.66 | -0.00 | 120,849 | 27,566 | Extendedly dominated |
|  | Ultrasound+AFP_full adherence (5) | 87,420 | 2,321 | 1.68 | 0.09 | 26,538 | 26,538 |  |
| 50-60 | Status quo (1) | 84,581 |  | 1.58 |  |  |  |  |
|  | Ultrasound_real-world adherence (2) | 85,999 | 1,418 | 1.63 | 0.05 | 29,091 | 29,091 | Extendedly dominated |
|  | Ultrasound+AFP_real-world adherence (3) | 86,440 | 441 | 1.65 | 0.02 | 24,464 | 27,843 | Extendedly dominated |
|  | Ultrasound_full adherence (4) | 86,445 | 5 | 1.65 | -0.00 | -13,496 | 28,085 | Absolutely dominated |
|  | Ultrasound+AFP_full adherence (5) | 86,933 | 2,352 | 1.67 | 0.09 | 26,965 | 26,965 |  |

*AFP; Alpha-Fetoprotein; HCC, hepatocellular carcinoma; ICER, Incremental cost-effectiveness ratio; Incr., incremental; QALY, quality adjusted life years*

† *A strategy with a higher ICER (relative to the next alternative) and lower QALY than the alternative was extendedly dominated by the alternative*

§ *A strategy with higher cost and lower QALY than the alternative was absolutely dominated by the alternative*


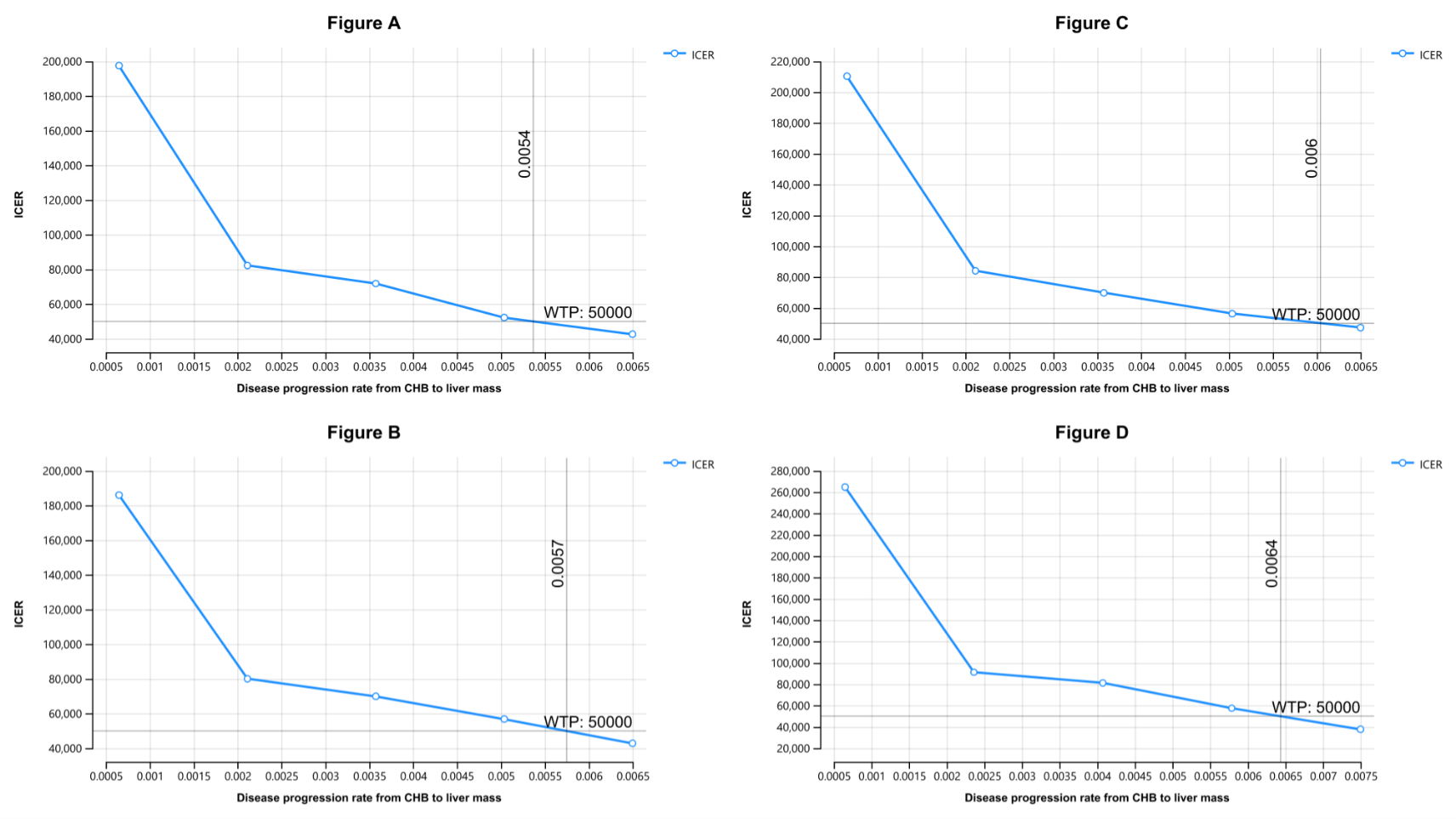
Appendix D. Results of sensitivity and threshold analyses


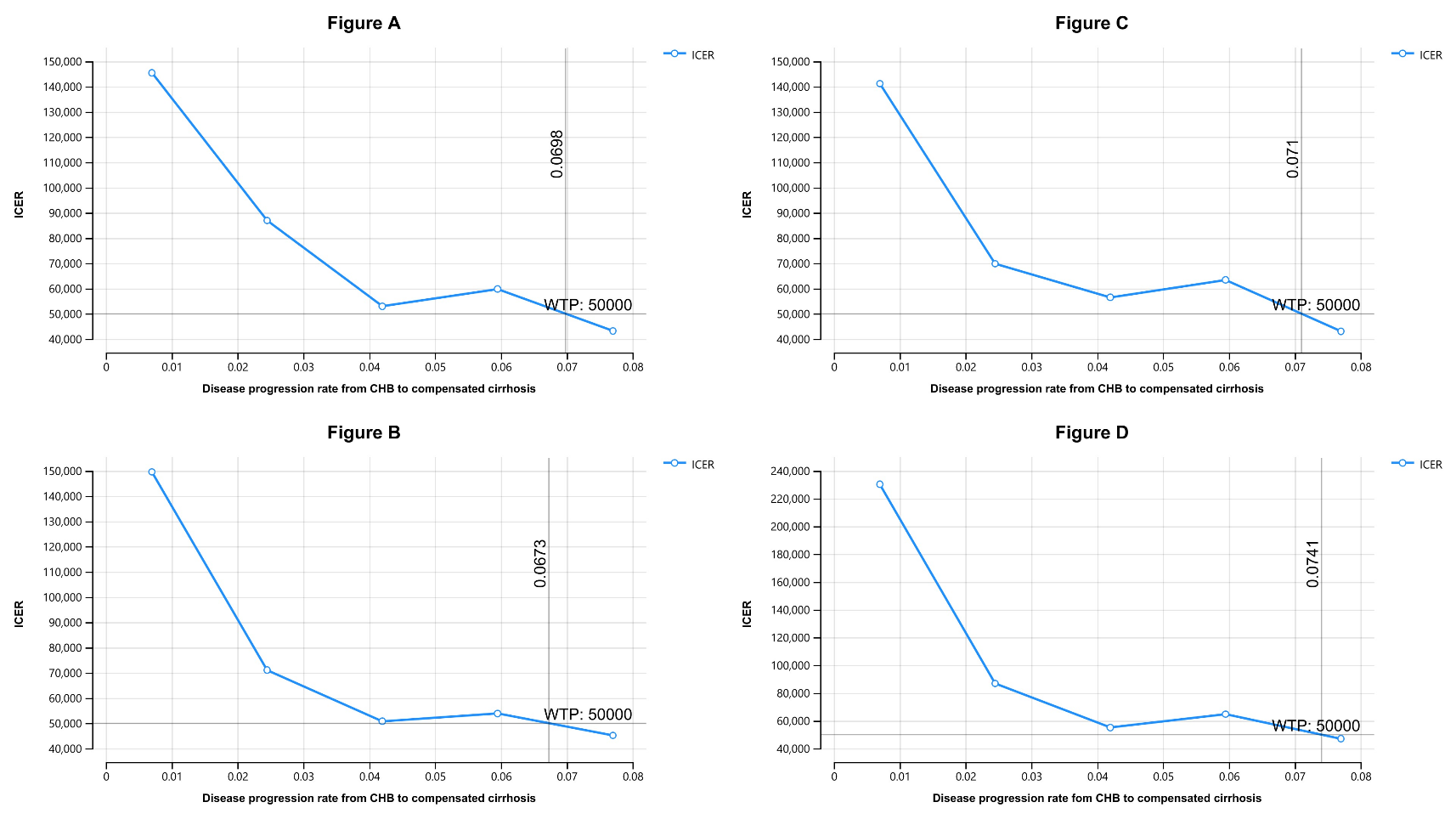
Figure D1. Disease progression rates from CHB to liver mass at different age ranges: (A) 20 to 80; (B) 30 to 80; (C) 40 to 80; (D) 50 to 80

Figure D2. Disease progression rates from CHB to compensated cirrhosis at different age ranges: (A) 20 to 80; (B) 30 to 80; (C) 40 to 80; (D) 50 to 80


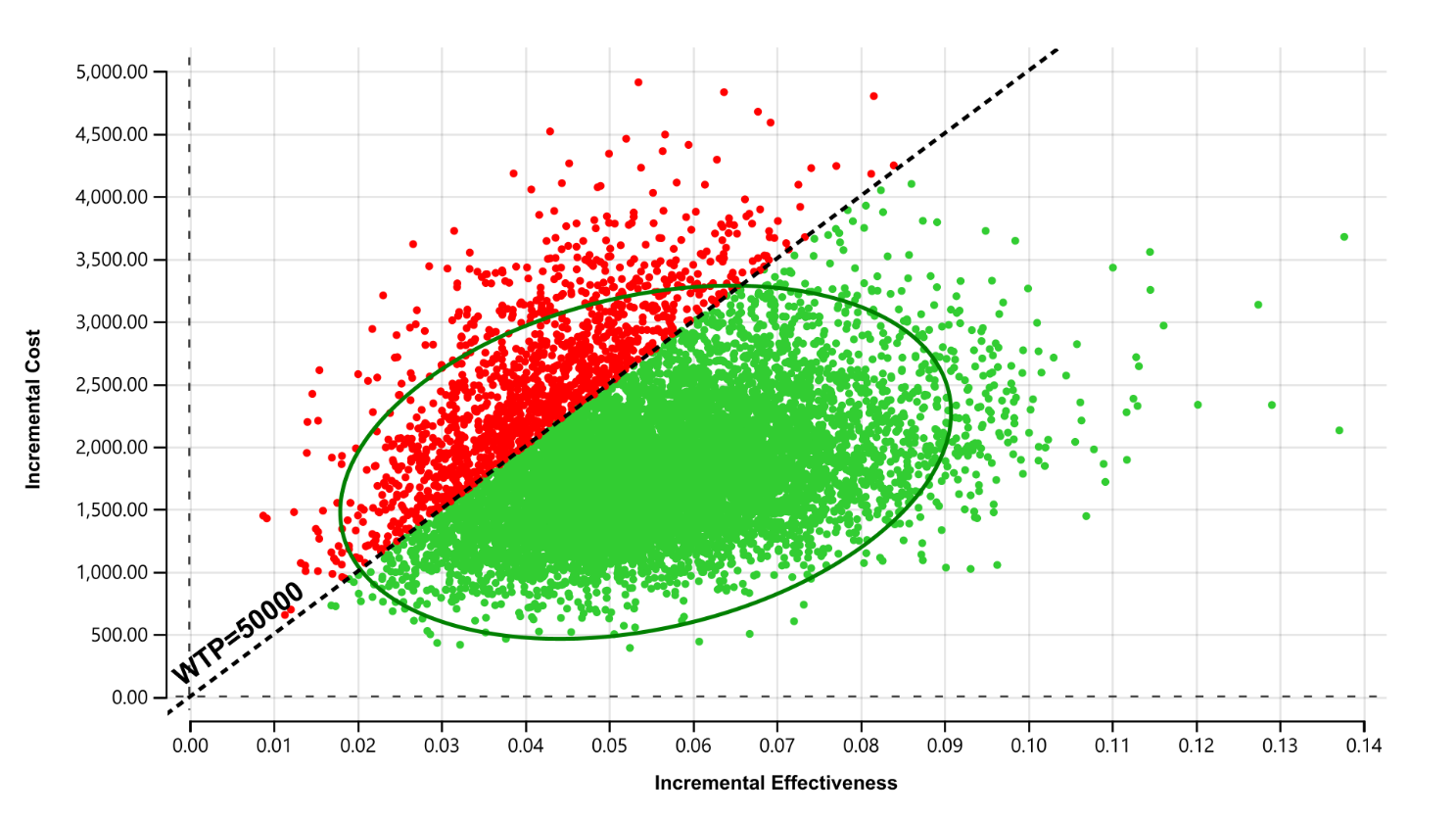
Figure D3. Incremental cost-effectiveness scatterplot for Ultrasound+AFP versus status quo at real-world adherence rates, baseline population aged 40 to 80 years (green points represent optimal strategies, eclipse area represents 95% confidence interval)


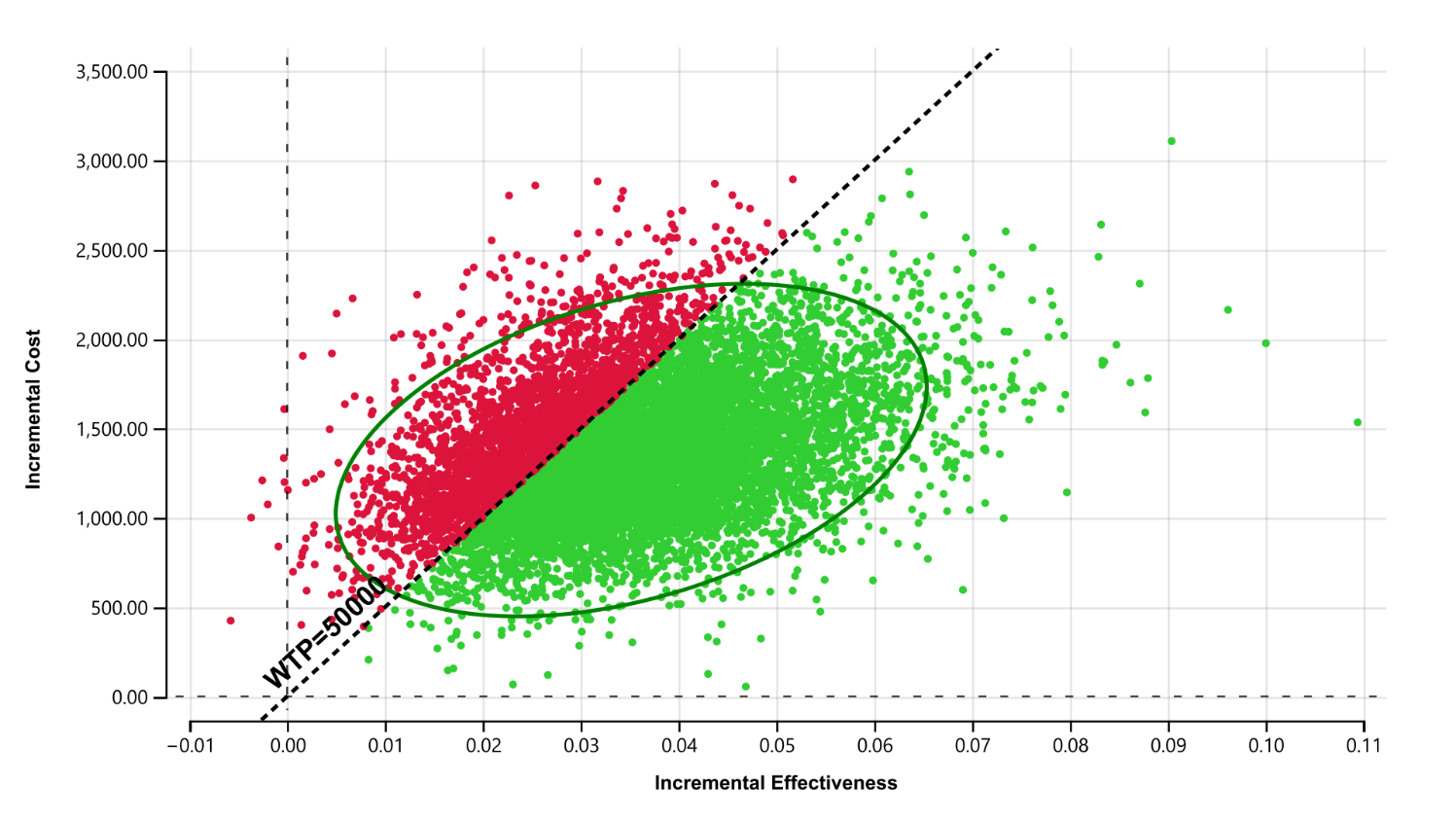


Figure D4. Incremental cost-effectiveness scatterplot for Ultrasound versus status quo at real-world adherence rate, baseline population aged 40 to 80 years (green points represent optimal strategies, eclipse area represents 95% confidence interval)

Appendix E. Validation of the model

Three types of validation was conducted for this model: face validity, internal validity and external validity, following the recommendation of the International Society for Pharmacoeconomics and Outcomes Research (ISPOR) [36]. For face validity, several meetings were conducted to demonstrate the documentation of the model during its building process and based on feedbacks from gastroenterologists, hepatologists and clinical nurse specialists, the model was improved. The model’s outcomes were compared with input data (internal validity) and published data not used in the model (external validity) for goodness-of-fit analyses. An ordinary least square linear regression line was fitted from the generated and observed data to compare with a 45° line reflecting perfect concordance, with the coefficient of determination (R^2^) generated to quantify how close the validation outcomes were to the OLS line. Four mean error estimates: Mean Absolute Percentage Error (MAPE) , Root Mean Squared Percentage Error (RMSPE), Mean Squared Log of the Accuracy Ratio (MSLAR) and Mean Squared Logarithmic Error (MSLE) were generated as part of the validation. The quality of model’s predicted outcomes increased with decreasing error estimates. Table E1 and E2 present the results from internal and external validation, respectively. Table E3 shows mean error values of internal and external validation and figure E shows the validity of the model

**Table E1.** Internal validity results

| Parameters | Predicted | Observed | Source |
| --- | --- | --- | --- |
| HCC incidence in compensated cirrhotic patients (per 100 person- years) | 2.509 | 2.200 | [3] |
| HCC incidence in decompensated cirrhotic patients (per 100 person- years) | 4.547 | 5.400 | [4] |
| HCC stages in population with no surveillance |  |  |  |
| Stage A | 0.094 | 0.096 | [14] |
| Stage B | 0.244 | 0.237 |  |
| Stage C | 0.499 | 0.500 |  |
| Stage D | 0.163 | 0.167 |  |
| HCC stages in population undergoing 6-monthly Ultrasound |  |  |  |
| Stage A | 0.495 | 0.491 | [14] |
| Stage B | 0.191 | 0.191 |  |
| Stage C | 0.177 | 0.182 |  |
| Stage D | 0.137 | 0.136 |  |
| HCC stages in population undergoing 6-monthly Ultrasound+AFP |  |  |  |
| Stage A | 0.622 | 0.618 | [15] |
| Stage B | 0.191 | 0.191 |  |
| Stage C | 0.122 | 0.129 |  |
| Stage D | 0.065 | 0.062 |  |
| Surveillance adherence rate |  |  |  |
| After 1 year | 0.672 | 0.672 | [16] |
| After 2 years | 0.470 | 0.470 |  |
| After 5 years | 0.400 | 0.400 |  |
| After 10 years | 0.239 | 0.239 |  |
| Lifetime liver transplant recurrence rate | 0.112 | 0.115 | [12] |
| Recurrence rate 5 years after Liver resection | 0.491 | 0.484 | [34] |
| Liver Transplant Perioperative probability of death | 0.034 | 0.035 | [12] |
| Liver Resection Perioperative probability of death | 0.044 | 0.048 | [13] |

**Table E2.** External validity results

| Parameters | Predicted | Observed | Source |
| --- | --- | --- | --- |
| Prevalence of HCC amongst Compensated Cirrhosis patients at 2 years of follow-up | 0.047 | 0.047 | [44] |
| 1-year survival rate amongst Compensated Cirrhosis patients | 0.950 | 0.873 | [45] |
| 1-year survival rate amongst Compensated Cirrhosis patients (40-64 years old) | 0.954 | 0.906 | [44] |
| 1-year survival rate amongst Compensated Cirrhosis patients (>64 years old) | 0.939 | 0.841 | [44] |
| 2-year survival rate amongst Compensated Cirrhosis patient (40-64 years old) | 0.894 | 0.847 | [44] |
| 2-year survival rate amongst Compensated Cirrhosis patients (>64 years old) | 0.870 | 0.759 | [44] |
| 5-year survival rate amongst Compensated Cirrhosis patients | 0.688 | 0.665 | [45] |
| 5-year survival rate amongst Compensated Cirrhosis patients (40-64 years old) | 0.706 | 0.721 | [44] |
| 5-year survival rate amongst Compensated Cirrhosis patients (>64 years old) | 0.644 | 0.576 | [44] |
| 1-year survival rate amongst Decompensated Cirrhosis patients | 0.810 | 0.750 | [45] |
| 1-year survival rate amongst Decompensated Cirrhosis patients (40-64 years old) | 0.813 | 0.651 | [44] |
| 1-year survival rate amongst Decompensated Cirrhosis patients (>64 years old) | 0.797 | 0.626 | [44] |
| 2-year survival rate amongst Decompensated Cirrhosis patient (40-64 years old) | 0.655 | 0.571 | [44] |
| 2-year survival rate amongst Decompensated Cirrhosis patients (>64 years old) | 0.629 | 0.524 | [44] |
| 5-year survival rate amongst Decompensated Cirrhosis patients | 0.315 | 0.454 | [45] |
| 5-year survival rate amongst Decompensated Cirrhosis patients (40-64 years old) | 0.323 | 0.417 | [44] |
| 5-year survival rate amongst Decompensated Cirrhosis patients (>64 years old) | 0.296 | 0.341 | [44] |
| 5-year survival rate of HCC | 0.203 | 0.209 | [46] |
| Rate of curative treatments for population with no surveillance | 0.357 | 0.237 | [47] |
| Rate of curative treatments for population undergoing 6-monthly Ultrasound | 0.514 | 0.516 | [47] |
| Rate of curative treatments for population undergoing 6-monthly Ultrasound+AFP | 0.561 |  | [47] |
| Sensitivities of ultrasound for early HCC detection (BCLC 0/A) | 0.489 | 0.450 | [48] |
| Sensitivities of ultrasound with AFP for early HCC detection (BCLC 0/A) | 0.612 | 0.630 | [48] |
| HCC incidence in CHB patients (per 100 person- years) | 0.384 | 0.300 | [3] |

**Table E3.** Mean error values of internal and external validation

|  | MAPE (%) | RMSPE (%) | MSLAR | MSLE |
| --- | --- | --- | --- | --- |
| Internal validation | 3.08 | 5.21 | 0.00 | 0.00 |
| External validation | 13.56 | 17.97 | 0.03 | 0.27 |
|  |  |  |  |  |


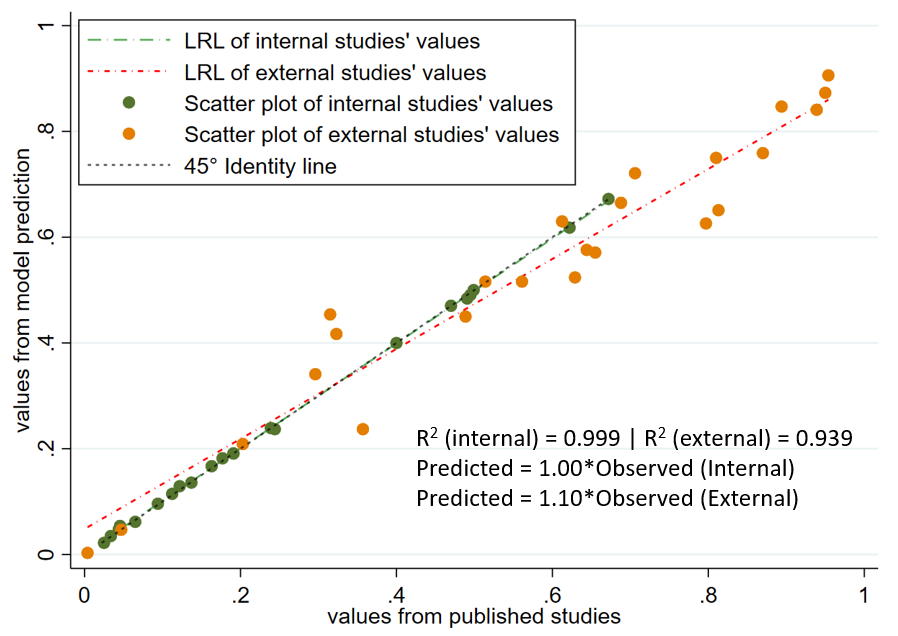


Figure E. Goodness-of-fit results of the model

Appendix F. Consolidated Health Economic Evaluation Reporting Standards (CHEERS) Checklist

| **Topic** | **No.** | **Item** | **Location where item is reported** |
| --- | --- | --- | --- |
| **Title** |  |  |  |
|  | 1 | Identify the study as an economic evaluation and specify the interventions being compared. | Page 1 |
| **Abstract** |  |  |  |
|  | 2 | Provide a structured summary that highlights context, key methods, results, and alternative analyses. | Page 1 |
| **Introduction** |  |  |  |
| **Background and objectives** | 3 | Give the context for the study, the study question, and its practical relevance for decision making in policy or practice. | Introduction section |
| **Methods** |  |  |  |
| **Health economic analysis plan** | 4 | Indicate whether a health economic analysis plan was developed and where available. | NA |
| **Study population** | 5 | Describe characteristics of the study population (such as age range, demographics, socioeconomic, or clinical characteristics). | Methods section, part 3 |
| **Setting and location** | 6 | Provide relevant contextual information that may influence findings. | Methods section, part 1; Introduction |
| **Comparators** | 7 | Describe the interventions or strategies being compared and why chosen. | Methods section, part 1 |
| **Perspective** | 8 | State the perspective(s) adopted by the study and why chosen. | Methods section, part 4.1 |
| **Time horizon** | 9 | State the time horizon for the study and why appropriate. | Methods section, part 2 |
| **Discount rate** | 10 | Report the discount rate(s) and reason chosen. | Methods section, part 4.1 |
| **Selection of outcomes** | 11 | Describe what outcomes were used as the measure(s) of benefit(s) and harm(s). | Methods section, part 4.2 |
| **Measurement of outcomes** | 12 | Describe how outcomes used to capture benefit(s) and harm(s) were measured. | Methods section, part 4.2 |
| **Valuation of outcomes** | 13 | Describe the population and methods used to measure and value outcomes. | Methods section, part 4.3 |
| **Measurement and valuation of resources and costs** | 14 | Describe how costs were valued. | Methods section, part 4.2 |
| **Currency, price date, and conversion** | 15 | Report the dates of the estimated resource quantities and unit costs, plus the currency and year of conversion. | Methods section, part 4.2 |
| **Rationale and description of model** | 16 | If modelling is used, describe in detail and why used. Report if the model is publicly available and where it can be accessed. | Methods section, part 2 |
| **Analytics and assumptions** | 17 | Describe any methods for analysing or statistically transforming data, any extrapolation methods, and approaches for validating any model used. | Methods section, part 6; Appendix E |
| **Characterising heterogeneity** | 18 | Describe any methods used for estimating how the results of the study vary for subgroups. | Methods section, part 3 & 6 |
| **Characterising distributional effects** | 19 | Describe how impacts are distributed across different individuals or adjustments made to reflect priority populations. | Methods section, part 3 |
| **Characterising uncertainty** | 20 | Describe methods to characterise any sources of uncertainty in the analysis. | Methods section, part 6 |
| **Approach to engagement with patients and others affected by the study** | 21 | Describe any approaches to engage patients or service recipients, the general public, communities, or stakeholders (such as clinicians or payers) in the design of the study. | Appendix E |
| **Results** |  |  |  |
| **Study parameters** | 22 | Report all analytic inputs (such as values, ranges, references) including uncertainty or distributional assumptions. | Appendix A |
| **Summary of main results** | 23 | Report the mean values for the main categories of costs and outcomes of interest and summarise them in the most appropriate overall measure. | Results section, table 1 |
| **Effect of uncertainty** | 24 | Describe how uncertainty about analytic judgments, inputs, or projections affect findings. Report the effect of choice of discount rate and time horizon, if applicable. | Table 2, Figure 2-4, Appendix C & D |
| **Effect of engagement with patients and others affected by the study** | 25 | Report on any difference patient/service recipient, general public, community, or stakeholder involvement made to the approach or findings of the study | Appendix E |
| **Discussion** |  |  |  |
| **Study findings, limitations, generalisability, and current knowledge** | 26 | Report key findings, limitations, ethical or equity considerations not captured, and how these could affect patients, policy, or practice. | Discussion section |
| **Other relevant information** |  |  |  |
| **Source of funding** | 27 | Describe how the study was funded and any role of the funder in the identification, design, conduct, and reporting of the analysis | Page 1 |
| **Conflicts of interest** | 28 | Report authors conflicts of interest according to journal or International Committee of Medical Journal Editors requirements. | Page 1 |

*From:* Husereau D, Drummond M, Augustovski F, et al. Consolidated Health Economic Evaluation Reporting Standards 2022 (CHEERS 2022) Explanation and Elaboration: A Report of the ISPOR CHEERS II Good Practices Task Force. Value Health 2022;25. <doi:10.1016/j.jval.2021.10.008>

Appendix G. Reference

1. Xiao, Y., et al., *Enhancing the hepatitis B care cascade in Australia: A cost-effectiveness model.* J Viral Hepat, 2020. **27**(5): p. 526-536.

2. D'Amico, G., G. Garcia-Tsao, and L. Pagliaro, *Natural history and prognostic indicators of survival in cirrhosis: a systematic review of 118 studies.* J Hepatol, 2006. **44**(1): p. 217-31.

3. Fattovich, G., F. Bortolotti, and F. Donato, *Natural history of chronic hepatitis B: special emphasis on disease progression and prognostic factors.* J Hepatol, 2008. **48**(2): p. 335-52.

4. Yang, M., et al., *Incidence and risk factors of hepatocellular carcinoma in patients with hepatitis C in China and the United States.* Sci Rep, 2020. **10**(1): p. 20922.

5. Parikh, N.D., et al., *Cost-Effectiveness of Hepatocellular Carcinoma Surveillance: An Assessment of Benefits and Harms.* Am J Gastroenterol, 2020. **115**(10): p. 1642-1649.

6. Farhang Zangneh, H., et al., *Cost Effectiveness of Hepatocellular Carcinoma Surveillance After a Sustained Virologic Response to Therapy in Patients With Hepatitis C Virus Infection and Advanced Fibrosis.* Clin Gastroenterol Hepatol, 2019. **17**(9): p. 1840-1849 e16.

7. Caturelli, E., et al., *Ultrasound guided fine needle biopsy of early hepatocellular carcinoma complicating liver cirrhosis: a multicentre study.* Gut, 2004. **53**(9): p. 1356-62.

8. Moctezuma-Velazquez, C., et al., *Non-invasive imaging criteria for the diagnosis of hepatocellular carcinoma in non-cirrhotic patients with chronic hepatitis B.* JHEP Rep, 2021. **3**(6): p. 100364.

9. Iavarone, M., et al., *Contrast imaging techniques to diagnose hepatocellular carcinoma in cirrhotics outside regular surveillance.* Ann Hepatol, 2019. **18**(2): p. 318-324.

10. Goossens, N., et al., *Cost-Effectiveness of Risk Score-Stratified Hepatocellular Carcinoma Screening in Patients with Cirrhosis.* Clin Transl Gastroenterol, 2017. **8**(6): p. e101.

11. Cabibbo, G., et al., *A meta-analysis of survival rates of untreated patients in randomized clinical trials of hepatocellular carcinoma.* Hepatology, 2010. **51**(4): p. 1274-83.

12. Australia & New Zealand Liver and Intestinal Transplant Registry, *31st ANZLITR Annual Report*, M.B. Michael Fink, Editor. 2019: Melbourne, Victoria, AUSTRALIA.

13. Gluer, A.M., et al., *Systematic review of actual 10-year survival following resection for hepatocellular carcinoma.* HPB (Oxford), 2012. **14**(5): p. 285-90.

14. Hong, T.P., et al., *Surveillance improves survival of patients with hepatocellular carcinoma: a prospective population-based study.* Medical Journal of Australia, 2018. **209**(8): p. 348-354.

15. van Meer, S., et al., *Surveillance for hepatocellular carcinoma is associated with increased survival: results from a large cohort in the Netherlands.* Journal of hepatology, 2015. **63**(5): p. 1156-1163.

16. Sarkar, M., et al., *Hepatocellular carcinoma screening practices and impact on survival among hepatitis B‐infected Asian Americans.* Journal of viral hepatitis, 2012. **19**(8): p. 594-600.

17. The Pharmaceutical Benefits Advisory Committee Guidelines, *Overview and rationale of the economic evaluation*.

18. MacLachlan, J., et al., *Viral hepatitis mapping project: national report 2018–19.* Darlinghurst, NSW, Australia: Australasian Society for HIV, Viral Hepatitis and Sexual Health Medicine (ASHM), 2020.

19. Tzartzeva, K., et al., *Surveillance Imaging and Alpha Fetoprotein for Early Detection of Hepatocellular Carcinoma in Patients With Cirrhosis: A Meta-analysis.* Gastroenterology, 2018. **154**(6): p. 1706-1718 e1.

20. Australian Department of Health, *Medicare Benefits Schedule Book - Operating from 1 January 2020*. 2020: Canberra ACT.

21. Cosic, L., et al., *The financial impact of postoperative complications following liver resection.* Medicine (Baltimore), 2019. **98**(27): p. e16054.

22. Independent Hospital Pricing Authority (IHPA). *National Hospital Cost Data Collection Report, Public Sector, Round 23 (Financial year 2018-19)*. 2021; Available from: <https://www.ihpa.gov.au/publications/national-hospital-cost-data-collection-report-public-sector-round-23-financial-year>.

23. The Pharmaceutical Benefits Scheme. *SORAFENIB price*. Available from: <https://www.pbs.gov.au/medicine/item/9380q>.

24. The Pharmaceutical Benefits Scheme. *LENVATINIB price*. Available from: <https://www.pbs.gov.au/medicine/item/10952K-11638M>.

25. Reeve, R., et al., *Health care use and costs at the end of life: a comparison of elderly Australian decedents with and without a cancer history.* BMC Palliative Care, 2018. **17**(1): p. 1-10.

26. McPhail, S.M., et al., *Assessment of health-related quality of life and health utilities in Australian patients with cirrhosis.* JGH Open, 2021. **5**(1): p. 133-142.

27. Kim, H.L., et al., *Magnetic resonance imaging is cost‐effective for hepatocellular carcinoma surveillance in high‐risk patients with cirrhosis.* Hepatology, 2019. **69**(4): p. 1599-1613.

28. Arguedas, M.R., et al., *Screening for hepatocellular carcinoma in patients with hepatitis C cirrhosis: a cost-utility analysis.* Am J Gastroenterol, 2003. **98**(3): p. 679-90.

29. Institute for Health Metrics and Evaluation (IHME), *Global Burden of Disease Study 2019 (GBD 2019) Disability Weights*. 2020: Seattle, United States of America.

30. Australian Institute of Health and Welfare. *Health Expenditure Australia 2019-20: Data tables for Health Expenditure Australia 2019-20*. 2021; Available from: <https://www.aihw.gov.au/reports/health-welfare-expenditure/health-expenditure-australia-2019-20/data>.

31. McCaffrey, N., et al., *Health-related quality of life measured using the EQ-5D–5L: South Australian population norms.* Health and quality of life outcomes, 2016. **14**(1): p. 1-12.

32. Yen, Y.H., et al., *Adherence to the modified Barcelona Clinic Liver Cancer guidelines: Results from a high-volume liver surgery center in East Asias.* PLoS One, 2021. **16**(3): p. e0249194.

33. DeOliveira, M.L., et al., *Cholangiocarcinoma: thirty-one-year experience with 564 patients at a single institution.* Annals of surgery, 2007. **245**(5): p. 755.

34. Tsilimigras, D.I., et al., *Recurrence patterns and outcomes after resection of hepatocellular carcinoma within and beyond the Barcelona Clinic Liver Cancer Criteria.* Annals of surgical oncology, 2020. **27**(7): p. 2321-2331.

35. Hung, H.H., et al., *Survival rates are comparable after radiofrequency ablation or surgery in patients with small hepatocellular carcinomas.* Clin Gastroenterol Hepatol, 2011. **9**(1): p. 79-86.

36. de'Angelis, N., et al., *Managements of recurrent hepatocellular carcinoma after liver transplantation: A systematic review.* World J Gastroenterol, 2015. **21**(39): p. 11185-98.

37. Chan, A.C., et al., *Treatment strategy for recurrent hepatocellular carcinoma: salvage transplantation, repeated resection, or radiofrequency ablation?* Liver Transpl, 2013. **19**(4): p. 411-9.

38. Doyle, A., et al., *Outcomes of radiofrequency ablation as first-line therapy for hepatocellular carcinoma less than 3 cm in potentially transplantable patients.* Journal of hepatology, 2019. **70**(5): p. 866-873.

39. Australian Bureau of Statistics. *National, state and territory population*. 2022; Available from: <https://www.abs.gov.au/statistics/people/population/national-state-and-territory-population/latest-release>.

40. Okinaga, H., et al., *Short-Term Outcomes following Hepatectomy in Elderly Patients with Hepatocellular Carcinoma: An Analysis of 10,805 Septuagenarians and 2,381 Octo- and Nonagenarians in Japan.* Liver Cancer, 2018. **7**(1): p. 55-64.

41. Sanyal, S., et al., *Outcomes of liver resection for hepatocellular carcinoma in octogenarians.* HPB (Oxford), 2020. **22**(9): p. 1324-1329.

42. Imamura, H., et al., *Feasibility and safety of surgical microwave ablation for hepatocellular carcinoma in elderly patients: a single center analysis in Japan.* Sci Rep, 2020. **10**(1): p. 14215.

43. Schullian, P., et al., *Stereotactic Radiofrequency Ablation of Liver Tumors in Octogenarians.* Front Oncol, 2019. **9**: p. 929.

44. Orman, E.S., et al., *Trends in Characteristics, Mortality, and Other Outcomes of Patients With Newly Diagnosed Cirrhosis.* JAMA Netw Open, 2019. **2**(6): p. e196412.

45. Fleming, K.M., et al., *All-cause mortality in people with cirrhosis compared with the general population: a population-based cohort study.* Liver Int, 2012. **32**(1): p. 79-84.

46. Australian Institute of Health and Welfare, *Cancer summary data visualisation.* 2021.

47. Singal, A.G., A. Pillai, and J. Tiro, *Early detection, curative treatment, and survival rates for hepatocellular carcinoma surveillance in patients with cirrhosis: a meta-analysis.* PLoS Med, 2014. **11**(4): p. e1001624.

48. Tzartzeva, K., et al., *Surveillance Imaging and Alpha Fetoprotein for Early Detection of Hepatocellular Carcinoma in Patients With Cirrhosis: A Meta-analysis.* Gastroenterology, 2018. **154**(6): p. 1706-1718.
